# A novel hypothesis-generating approach for detecting phenotypic associations using epigenetic data

**DOI:** 10.1080/17501911.2024.2366157

**Published:** 2024-07-17

**Authors:** Florence Z Martin, Kayleigh E Easey, Laura D Howe, Abigail Fraser, Deborah A Lawlor, Caroline L Relton, Gemma C Sharp

**Affiliations:** a MRC Integrative Epidemiology Unit at the University of Bristol, Bristol, UK; b Population Health Sciences, Bristol Medical School, Bristol, UK; c School of Psychological Science, University of Bristol, Bristol, UK; d London School of Hygiene & Tropical Medicine, London, UK; e School of Psychology, University of Exeter, Exeter, UK

**Keywords:** ALSPAC, ARIES, dysmenorrhea, epigenome-wide association study, heavy menstrual bleeding, hypothesis-generating, hypothesis-testing

## Abstract

**Aim:** Hypotheses about what phenotypes to include in causal analyses, that in turn can have clinical and policy implications, can be guided by hypothesis-free approaches leveraging the epigenome, for example.

**Materials & methods:** Minimally adjusted epigenome-wide association studies (EWAS) using ALSPAC data were performed for example conditions, dysmenorrhea and heavy menstrual bleeding (HMB). Differentially methylated CpGs were searched in the EWAS Catalog and associated traits identified. Traits were compared between those with and without the example conditions in ALSPAC.

**Results:** Seven CpG sites were associated with dysmenorrhea and two with HMB. Smoking and adverse childhood experience score were associated with both conditions in the hypothesis-testing phase.

**Conclusion:** Hypothesis-generating EWAS can help identify associations for future analyses.

## Plain Language Summary

To inform policy and improve clinical practice, it is important that researchers who study people's health find out which traits might increase the risk of illness. However, it can be difficult to know which traits should be looked at. In this study, we wanted to look for traits that might increase the risk of painful and heavy periods, using data about the switches that turn our genes on and off. There are some people in the Children of the 90s study that have data on gene switches. We compared all the switches between those with and without painful or heavy periods. For painful periods, we found links with seven switches and for heavy periods, we found two. We then used another data source, called the EWAS Catalog, to see which traits were associated with these switches. The traits we found included body size, smoking and child abuse. Finally, when using data on traits from the wider Children of the 90s group, we found that smoking and more difficult childhoods were some of the traits related to painful and heavy periods. A good thing about this approach is that we could find new traits that might increase the risk of painful or heavy periods; these should be looked at in future studies.

## Tweetable Abstract

Leveraging EWAS data can identify novel risk factors for conditions such as menstrual symptoms in future, causally motivated analyses: a proof-of-concept study in the Children of the 90's cohort (ALSPAC)

## Abstract (plain language summary)

**Background**

- DNA methylation can be explored in an epigenome-wide association study (EWAS) context to identify causal mechanisms or confounding relationships between exposures that can alter the epigenome and phenotypes of interest.
- In a minimally adjusted EWAS acting as hypothesis-generating, associations can be identified that represent either one of these relationships to be further explored in causally motivated analyses for conditions that are understudied.
- In the present study, we demonstrated the utility of a hypothesis-generating EWAS approach followed by a hypothesis-testing phase using logistic regression, investigating two understudied conditions as examples: dysmenorrhea (painful periods) and heavy menstrual bleeding (HMB).

We used the Avon Longitudinal Study of Parents And Children (ALSPAC) to identify cases of adolescent dysmenorrhea and HMB; those with epigenetic data from ARIES were included in two hypothesis-generating EWAS where each condition served as the exposure.

The hypothesis-generating phase consisted of two minimally adjusted EWASs of dysmenorrhea and HMB to identify differentially methylated CpGs.

CpGs identified in the hypothesis-generating phase were then searched in the EWAS Catalog to find associated traits with the CpG and its resident gene.

The hypothesis-testing phase involved taking identified traits and investigating their association with dysmenorrhea and HMB in the wider ALSPAC cohort.

Having found seven differentially methylated CpGs for dysmenorrhea and two for HMB, we searched them in the EWAS Catalog and identified phenotypes associated with each of them.

In the hypothesis-testing phase, we proxied the phenotypes found in the EWAS Catalog using variables from ALSPAC and included them in minimally adjusted logistic regression models where each condition served as the outcome.

Using this approach, we found that smoking and alcohol use at age 13 was associated with dysmenorrhea and HMB; higher cotinine levels at age 7 was associated with HMB. Higher adverse childhood experience (ACE) score was associated with both conditions.

We identified several potential targets of investigation for future research into risk factors for dysmenorrhea and HMB. Although temporality was not easily established in the present study and causality indeterminable, we leverage confounding to guide future causally motivated analyses in other cohorts with menstruation data.

## 1. Background

Epigenome-wide association studies (EWAS) have been widely used in epidemiology over the last decade to explore biomarkers and etiologies of health traits and disease [1]. This explosion in use is due to both the volume and nature of the epigenetic data available to researchers, thanks to advances in bead-based microarray technology to measure levels of DNA methylation (DNAm) at individual CpG sites [2]. The epigenome is a dynamic system of mitotically heritable markers that can control gene expression without changing the underlying genetic sequence; it can be altered by environmental exposures associated with a multitude of phenotypes [3]. Traditional DNAm-based EWAS analyses aim to identify phenotype-CpG associations, under the assumption that these associations may either be causal (i.e., the CpG causes the phenotype or *vice versa*) or represent confounding in a way that could still be useful for indicating (historical) exposures or predicting future outcomes [4] (e.g., methylation at *AHRR* can be used to identify current and former smokers, and is predictive of lung cancer, even in the absence of causal mediation [5]). DNAm at specific CpGs can be thought of as phenotypes, because their variation have both a genetic and an environmental basis [6]. This feature means that, even in the absence of any causal epigenetic relationship, DNAm data may be useful for identifying (potentially causal) associations between other, non-epigenetic phenotypes, which can be followed by more causally motivated analyses. This kind of hypothesis generation is particularly useful for under-researched phenotypes and conditions where limited previous literature is available to guide hypothesis-driven approaches.

Menstrual health is one such example of an under-researched area. For reasons related to entrenched gender inequalities and menstrual stigma [7], there are still knowledge gaps around the risk factors and consequences of experiencing problematic menstrual symptoms like menstrual pain (dysmenorrhea) and heavy (or prolonged) menstrual bleeding (HMB), both of which are difficult to quantify and diagnose. However, these are common symptoms that affect a large proportion of the menstruating population throughout the life course that may impact on day-to-day life [8]; these symptoms can also be considered important indicators of other domains of health and wellbeing [9]. The prevalence of these in adolescence is estimated to be between 43% to 93% for dysmenorrhea [10] and 37% for HMB [11]. Behaviours such as smoking and characteristics such as body mass index (BMI) have been linked inconclusively to dysmenorrhea, with a number of – but not all – studies reporting associations [12–15]. The picture is even less clear for HMB outside comorbidities such as ovulatory dysfunction and coagulation disorders [16], with potential links to high BMI, smoking and alcohol consumption [17].

In this study, we aimed to use a minimally adjusted EWAS to leverage confounding and identify phenotypes that may be associated with dysmenorrhea and HMB as example conditions, where associations were tested in the wider cohort data in a later phase. In this paper, we use the term phenotype to refer to any non-genetic characteristic that might be a potential risk factor. The approach, in short, involved: identifying condition-CpG associations by running EWAS in the Avon Longitudinal Study of Parents and Children (ALSPAC) among those (G1) adolescents with epigenetic data; looking up identified CpGs in an online repository of published EWAS results (EWAS Catalog [18]) to identify phenotypes associated with those CpGs (i.e., generating hypotheses about phenotypic associations); and testing those hypotheses in the full ALSPAC cohort to explore associations between our conditions (dysmenorrhea and HMB) and the EWAS Catalog phenotypes (i.e., testing hypotheses). The two conditions under investigation here are understudied and prevalent, with little known about modifiable risk factors, thus making them useful case studies for proposed utility of the hypothesis-generating EWAS approach.

## 2. Materials & methods

### 2.1. Study population

ALSPAC is a longitudinal birth cohort study that recruited pregnant women between 1990 and 1992 in the previous area of Avon, in the Southwest of England [19,20] . The initial sample consisted of 14,541 pregnancies enrolled in the first phase; a further 906 pregnancies were added at subsequent recruitment phases, leading to 14,901 index children remaining in the cohort after 1 year [19,20] . A subset of 1,018 ALSPAC mother-child pairs, selected based on the availability of DNA samples, was included in the Accessible Resource for Integrative Epidemiologic Studies (ARIES) study, which generated DNA methylation data at three timepoints: birth, childhood and adolescence [21].

**Figure 1.**
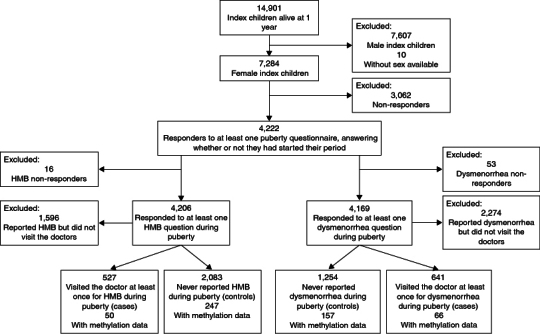
Flow of participants through the study. HMB: Heavy menstrual bleeding.

The initial eligibility criteria for this study were female index children (G1 participants) who had responded to at least one of the nine “G1 puberty” questionnaires sent between the ages of 8 and 17 years, specifically the section pertaining to menstruation (whether it had started, whether there were issues associated with it, etc.). Among those who fulfilled the initial eligibility criteria, we then identified those who had participated in ARIES and had methylation data (described below). The exclusions are summarised in the flow diagram (Figure 1).

Ethical approval for the study was obtained from the ALSPAC Ethics and Law Committee and the Local Research Ethics Committee. Informed consent for the use of data collected via questionnaires and clinics was obtained from participants following the recommendations of the ALSPAC Ethics and Law Committee at the time. Consent for biological samples was collected in accordance with the Human Tissue Act 2004. For data collection from participants at 22 years old and onwards, study data were collected and managed using REDCap (Research Electronic Data Capture) electronic data capture tools hosted at the University of Bristol [22]. The ALSPAC study website contains details of all the data that is available through a fully searchable data dictionary and variable search tool (http://www.bristol.ac.uk/alspac/researchers/our-data/) [23].

### 2.2. Definition of menstrual symptoms

In each of the nine G1 puberty questionnaires, the participants' caregiver (age 8–13) or participant (age 14 and over) was asked “Have you/your daughter ever had any of the following symptoms associated with your/her period: Severe cramps?” and “Heavy or prolonged bleeding?”. Answering “Yes” to each question prompted a question asking if a doctor was contacted for the symptom. Other than at age 15 for cramps, we did not have self-reported severity for either symptom, so for the purpose of the current study, we used “ever having visited the doctor” to define dysmenorrhea or HMB. Those who never reported to have experienced the symptom (or having only reported mild cramps at age 15 for dysmenorrhea) were designated controls. The menstrual health-related data in ALSPAC is summarised in detail elsewhere [24].

### 2.3. Identifying novel traits associated with menstrual symptoms

**Figure 2.**
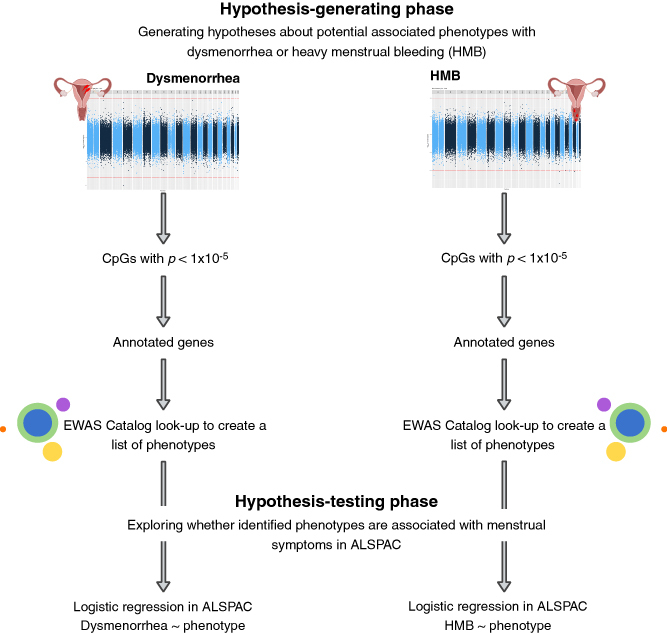
Our methodology for identifying novel phenotypes associated with dysmenorrhea and heavy menstrual bleeding in adolescents using a hypothesis-generating epigenome-wide association study approach. Created with BioRender.com.

The approach is detailed in Figure 2 and below.

#### 2.3.1. Hypothesis-generating phase

Generation and preparation of the DNA methylation data for ARIES is described in detail in the ARIES Data Resource Profile [21]. Briefly, DNA methylation in peripheral blood samples obtained from 1018 adolescents (aged 15 or 17 years old) was measured using the Illumina Infinium® HumanMethylation450K BeadChip assay [21]. This array contains probes that can measure the methylation of over 450,000 CpGs located throughout the human genome, driven by DNA bisulfite conversion [25]. The level of methylation is given as a beta-value (*b*), ranging from not methylated (*b* = 0) to fully methylated (*b* = 1) [26].

Data obtained from the array were pre-processed, normalised and quality controlled using the R package meffil [27]. We removed 11,648 probes that mapped to either the X or Y chromosomes (due to lack of information available in the EWAS Catalog as sex chromosomes removal is common practice in EWAS analyses), 901 SNP and control probes and 3853 probes that had a high detection *p*-value. Following the removal of these probes, samples with outlying methylation values were identified using the Tukey method (outside the 25th and 75th percentiles ±three-times the interquartile range) [28] and removed for individual probes. To account for cross-reactive probes and polymorphic CpGs, as guided by Chen et al., we retained all remaining probes and then checked the results against their list so as not to misinterpret spurious signals [29]. In the final analysis, 470,334 probes remained.

#### 2.3.2. Epigenome-wide association studies: hypothesis-generating phase

The relationships between methylation and menstrual symptoms were explored using a cross-sectional design within ALSPAC (i.e., both DNA methylation and menstrual symptoms were measured at ages that overlapped). Association between dysmenorrhea/HMB case/control status and variation in methylation was assessed using linear regression. Case status (dysmenorrhea or HMB any time during adolescence) was treated as the exposure in the EWAS, with methylation included as the outcome, since our aim was not to identify causal associations. Most dysmenorrhea (*n* = 65) and HMB (*n* = 46) cases reported to have experienced the symptom prior to their methylation being measured, either at 15 or, for the majority of ARIES participants, 17 years old. The small number of participants (*n* = 5) whose methylation was measured before the first reporting of their menstrual symptom were dealt with in sensitivity analysis (described below).

Models were purposefully simple: we adjusted for age at methylation measurement (given that the adolescent peripheral blood samples were collected at either age 15 or 17) and surrogate variables (SVs) generated to capture technical batch effects only. SVs were generated using SV analysis (SVA) and the number of SVs to generate was estimated as part of the SVA pipeline, based on the dataset (*nSV* = 24 and *nSV* = 33 for dysmenorrhea and HMB EWAS, respectively). All analyses were performed using the R package meffil (which draws on the R package isva to generate surrogate variables) [27] using R version 3.6.3. To enable the methylation data to capture variation in a wide range of other traits, we performed no further adjustment, e.g., for cell counts or hormonal contraception. Although adjustment for cell composition is standard in other epigenetic studies where CpG methylation is a cause of interest, we did not want to mask any generated hypotheses about cell composition, such as immune dysregulation, by adjusting for it.

#### 2.3.3. EWAS catalog look up

From each EWAS (of dysmenorrhea and HMB), we selected differentially methylated CpGs with a *p*-value <1 × 10^-5^ and performed a look-up of these CpGs, or the genes they mapped to, in the EWAS Catalog [18]. The EWAS Catalog is a repository of phenotype-CpG associations identified through published EWAS. We created a list of phenotypes associated with these CpGs and their resident gene; although pleiotropy was present for some CpGs, an enrichment analysis was not deemed necessary due to the hypothesis-generating nature of the analysis.

#### 2.3.4. Hypothesis-testing phase in full alspac sample

We used the ALSPAC data dictionary to find data on corresponding CpG-associated phenotypes in ALSPAC, assessed during gestation (for prenatal exposures) or pre-puberty (for childhood traits). Pre-puberty measurements of traits were chosen to determine whether the identified phenotype preceded the onset of these symptoms, and thus may be a candidate for future testing in causal analyses in other datasets as potential risk factors for either menstrual symptom. Further details of this process are described in the Supplementary Material. To explore the phenotypes identified in the look up phase, we performed logistic regressions with each identified phenotype included in turn as the exposure and the menstrual symptoms (dysmenorrhea or HMB) in ALSPAC adolescents as the outcome, with and without adjustment for socioeconomic position (SEP) and age at menarche (AAM). SEP has been shown to be associated with both menstrual symptoms [30–33] and most phenotypes; younger AAM is a risk factor for both menstrual symptoms [34] and is likely associated with several factors in adolescence included in the hypothesis-testing phase. Continuous variables were converted to standardized z-scores before running these analyses to enable comparison of effect estimates for variables on different scales. Phenotypes where numbers of cases and/or controls contained fewer than five participants were omitted from the analysis due to inadequate power.

To test previously identified associations between phenotypes such as gynecological and endocrine disorders, socioeconomic position, contraception and age at menarche and menstrual symptoms, we also included these alongside the series of logistic regressions of novel associations.

#### 2.3.5. Sensitivity analyses

We ran the EWAS analysis for each symptom again removing cases of thyroid problems (self-reported at 17 years), polycystic ovary syndrome (PCOS) and endometriosis (self-reported at 22 years) to limit the effect these conditions might have had on the findings. We also replicated the hypothesis-testing phase among participants who had reported the symptom during puberty but had not visited the doctor, to identify whether any characteristics were associated with a less “severe” presentation of dysmenorrhea or HMB and excluding those who first reported either symptom after their methylation was measured for ARIES. We investigated use of oral contraception in the hypothesis-testing phase rather than excluding them given the high prevalence of use among adolescents.

## 3. Results

Of the original ALSPAC cohort, 7284 participants were female (49% of the children who were alive at 1 year old) and 4222 of these participants responded to at least one of the questionnaires sent out during puberty, stating whether they had started their period. Of these, 487 individuals had DNA methylation data at adolescence in ARIES (Figure 1) and were included in the hypothesis-generating phase (QQ plots available in Supplementary Figures S1 & S2). In the hypothesis-testing phase, we performed complete case analyses, so the denominator differed by regression model depending on the missing data for each phenotype (Table 1).

**Table 1.** Number of each binary (*n*, %) and continuous characteristics (mean, standard deviation) in cases and controls for each symptom identified in the hypothesis-generating EWAS and missing data in each variable, where G0 refers to mums and G1 refers to adolescents.

	Dysmenorrhea		HMB	
	G1 cases n (%) (unless otherwise specified)	G1 controls n (%) (unless otherwise specified)	G1 cases n (%) (unless otherwise specified)	G1 controls n (%) (unless otherwise specified)
	641	1,254	527	2,083
Binary characteristics				
Prenatal				
Maternal (G0) university degree^†^	70 (10.9)	208 (16.6)	46 (8.7)	361 (17.3)
Missing	72 (11.2)	137 (10.9)	65 (12.3)	188 (9.0)
G0 alcohol consumption during pregnancy	394 (61.5)	785 (62.6)	317 (60.2)	1,336 (64.1)
Missing	151 (23.6)	258 (20.6)	129 (24.5)	397 (19.1)
G0 smoking during pregnancy	142 (22.2)	222 (17.7)	132 (25.0)	364 (17.5)
Missing	95 (14.8)	184 (14.7)	86 (16.3)	282 (13.5)
G0 hypertensive disorder of pregnancy (HDP)	113 (17.6)	201 (16.0)	97 (18.4)	310 (14.9)
Missing	44 (6.9)	67 (5.3)	41 (7.8)	104 (5.0)
G0 pre-eclampsia	9 (1.4)	26 (2.1)	11 (2.1)	42 (2.0)
Missing	44 (6.9)	67 (5.3)	41 (7.8)	104 (5.0)
Age 13 years				
G1 has drunk alcohol before	165 (25.7)	242 (19.3)	137 (26.0)	430 (20.6)
Missing	311 (48.5)	649 (51.8)	240 (45.5)	1,013 (48.6)
G1 has smoked cigarettes before^†^	99 (15.4)	92 (7.3)	91 (17.3)	160 (7.7)
Missing	160 (25.0)	383 (30.5)	130 (24.7)	544 (26.1)
End of puberty				
G1 oral contraception use^†^	445 (69.4)	271 (21.6)	390 (74.0)	511 (24.5)
Missing	<5	7 (0.6)	<5	11 (0.005)
G1 comorbidity^‡^ reported^†^	39 (6.1)	30 (2.4)	36 (6.8)	58 (2.8)
Missing	363 (56.6)	837 (66.7)	301 (57.1)	1292 (62.0)
Continuous characteristics				
Prenatal				
Mean G0 body mass index (BMI) during pregnancy, kg/m^2^ (SD)	23.1 (3.9)	22.9 (3.9)	23.1 (4.0)	22.8 (3.7)
Missing	94 (14.7)	201 (16.0)	80 (15.1)	301 (14.4)
Delivery				
Mean G1 gestational age at delivery, weeks (SD)	39.6 (1.7)	39.4 (1.8)	39.5 (1.8)	39.6 (1.8)
Missing	39 (6.1)	64 (5.1)	35 (6.6)	100 (4.8)
Age 7 years				
Mean G1 BMI, kg/m^2^ (SD)	16.8 (2.6)	16.3 (2.1)	16.8 (2.5)	16.3 (2.1)
Missing	137 (21.4)	318 (25.4)	114 (21.6)	450 (21.6)
Mean G1 cholesterol, mmol/l (SD)	4.5 (0.7)	4.5 (0.7)	4.5 (0.7)	4.5 (0.7)
Missing	309 (48.2)	636 (50.7)	253 (48.0)	1,008 (48.4)
Mean G1 cotinine, ng/ml (SD)	1.5 (1.4)	1.2 (1.2)	1.5 (1.5)	1.2 (1.1)
Missing	302 (48.2)	617 (49.2)	254 (48.2)	978 (47.0)
Age 8 years				
Mean G1 non-word repetition score, (SD)	7.2 (2.5)	7.3 (2.6)	7.1 (2.4)	7.4 (2.5)
Missing	62 (9.7)	368 (29.3)	145 (27.5)	527 (25.3)
Age 9 years				
Mean G1 C-reactive protein, mmol/l (SD)	1.2 (3.7)	0.7 (1.4)	1.2 (3.2)	0.8 (2.7)
Missing	322 (50.2)	625 (49.8)	279 (52.9)	981 (47.1)
Puberty				
Mean G1 age at menarche, months (SD)^†^	146.0 (12.8)	151.5 (12.6)	146.4 (13.3)	150.4 (12.3)
Missing	123 (19.2)	331 (26.4)	91 (17.3)	478 (22.9)
Age 16 years				
Mean G1 adverse childhood experience (ACE) score (SD)	2.0 (1.5)	1.6 (1.7)	2.1 (1.9)	1.5 (1.5)
Missing	419 (65.4)	892 (71.1)	362 (68.7)	1,433 (68.8)

† Identified characteristic *a priori*.

‡ Thyroid problems, PCOS, endometriosis.

### 3.1. Hypothesis-generating phase

#### 3.1.1. Dysmenorrhea

We identified seven differentially methylated CpG sites (*p* <1 × 10^-5^) (Table 2) in adolescents who suffered from severe dysmenorrhea compared with those who did not. None of these CpGs are represented in the Chen list of cross-reactive or polymorphic probes. DNA methylation at these CpG sites was associated with 9 phenotypes, and the genes they sit in were associated with a further 22 phenotypes in the EWAS Catalog look-up.

**Table 2.** Differentially methylated CpG sites identified in the hypothesis-generating EWAS of dysmenorrhea.

Probe ID	β (95%CI)	*p*-value	Probe position	CpG traits	Gene	Gene traits	Ref.
cg08142094	-0.036 (-0.050 to -0.022)	1.02 × 10^-6^	Chr16 85731256	–	–	–	
cg23012731	-0.008 (-0.011 to -0.005)	3.53 × 10^-6^	Chr1 33438978	–	–	–	
cg21802726	-0.002 (-0.003 to -0.004)	3.74 × 10^-4^	Chr20 33735257	Rheumatoid arthritis	*EDEM2*	Rheumatoid arthritis, ischaemic stroke, primary Sjögren's syndrome, HIV infection	[35,36]
cg04583842	-0.034 (-0.053 to -0.016)	4.17 × 10^-4^	Chr16 88103117	Smoking, gestational age, BMI, CRP, mortality	*BANP*	Clear cell renal carcinoma, fetal vs adult liver, HIV infection, smoking, age, gestational age, rheumatoid arthritis, BMI, primary Sjögren's syndrome, chronic kidney disease, CRP, sex, mortality, child abuse, melanoma, alcohol consumption, Crohn's disease, pre-eclampsia, air pollution exposure, maternal urinary arsenic, cognitive ability, total cholesterol chylomicrons and extremely large vLDLs, FASD	[35–45]
cg22603569	-0.017 (-0.029 to -0.005)	6.14 × 10^-3^	Chr19 3388047	Fetal vs adult liver, gestational age, alcohol consumption, hypertensive disorders of pregnancy (HDPs)	*NFIC*	Fetal vs adult liver, gestational age, smoking, pancreatic ductal adenocarcinoma, aging, rheumatoid arthritis, cleft lip vs palate, age, HIV infection, alcohol consumption, primary Sjögren's syndrome, sex, time spent sitting, pre-eclampsia HDPs, Crohn's disease	[35–38,42–44]
cg04737758	0.042 (0.025 to 0.058)	1.13 × 10^-6^	Chr14 74824136	–	–	–	
cg15017604	0.026 (0.015 to 0.037)	9.39 × 10^-6^	Chr11 1361518	–	–	–	

Probe ID denotes the differentially methylated CpG site. β represents the extent of methylation, with respective *p*-values. Probe position represents the chromosomal location of the CpG site, while CpG traits denotes traits that have been identified to be associated with that CpG site. The gene column shows in which gene the CpG site in question resides, with gene traits representing traits that have been shown to be associated with CpG sites located within that gene.

#### 3.1.2. HMB

In the EWAS of HMB, we identified two differentially methylated CpG sites with *p* <1 × 10^-5^ (Table 3). Similarly to dysmenorrhea, neither of these CpGs are represented in the Chen list. When we performed a look-up of these CpGs, as well as the genes they mapped to, in the EWAS Catalog, we identified 10 associated phenotypes.

**Table 3.** Differentially methylated CpG sites identified in the hypothesis-generating EWAS of heavy menstrual bleeding.

Probe ID	β (95% CI)	*p*-value	Probe position	CpG traits	Gene	Gene traits	Ref.
cg24196053	-0.010 (-0.014 to -0.006)	1.50 × 10^-5^	Chr4 1736433	–	*TACC3*	Clear cell renal carcinoma, gestational age, HIV infection, Crohn's disease, Alzheimer's disease, pre-eclampsia, total cholesterol	[38,43,44]
cg11465939	-0.029 (-0.042 to -0.015)	6.04 × 10^-5^	Chr22 23923462	Fetal vs adult liver, smoking, gestational age	*IGLL1*	Clear cell renal carcinoma, fetal vs adult liver, gestational age, HIV infection, primary Sjögren's syndrome, smoking	[36–38]

Probe ID denotes the differentially methylated CpG site. β represents the extent of methylation, with respective *p*-values. Probe position represents the chromosomal location of the CpG site, while CpG traits denotes traits that have been identified to be associated with that CpG site. The gene column shows in which gene the CpG site in question resides, with gene traits representing traits that have been shown to be associated with CpG sites located within that gene.

### 3.2. Hypothesis-testing phase

From the list of phenotypes that were associated with differentially methylated CpGs in the hypothesis-generating phase (Tables 2 & 3), we derived the following variables from ALSPAC: maternal educational attainment, maternal smoking and alcohol consumption during pregnancy, maternal BMI, maternal hypertensive disorders of pregnancy (HDP), maternal pre-eclampsia, participant gestational age at delivery, participant BMI, cotinine and cholesterol levels at age 7, participant non-word repetition score (measure of cognition) at age 8, participant C-reactive protein (CRP) at age 9, participant cigarette and alcohol use at age 13, participant adverse childhood experience (ACE) score by 16 years old [46] and oral contraception use during puberty (derived from the G1 puberty questionnaires that asked specifically about oral contraception, detailed in the Supplementary). Of these, participant smoking and BMI had been identified *a priori* as associated with both menstrual symptoms. We then compared these variables for cases and controls for each condition in the wider ALSPAC cohort (*n* = 4,222) Other characteristics such as kidney disease, primary Sjögren's syndrome, melanoma, Crohn's disease and rheumatoid arthritis were available, but cases and/or controls for each symptom contained fewer than five participants so were omitted from the analysis as described in the methods.

**Figure 3.**
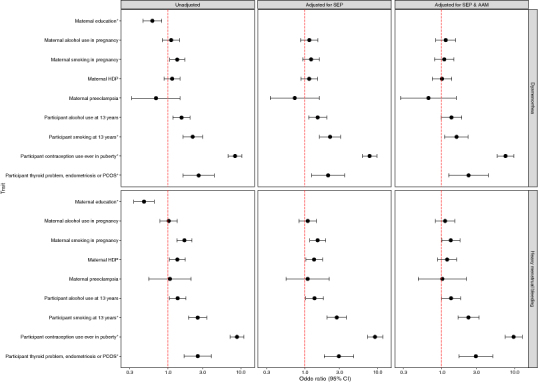
Coefficient plot representing binary phenotypes associated with dysmenorrhea and heavy menstrual bleeding in the hypothesis testing phase. * Identified as an associated trait *a priori*.

**Figure 4.**
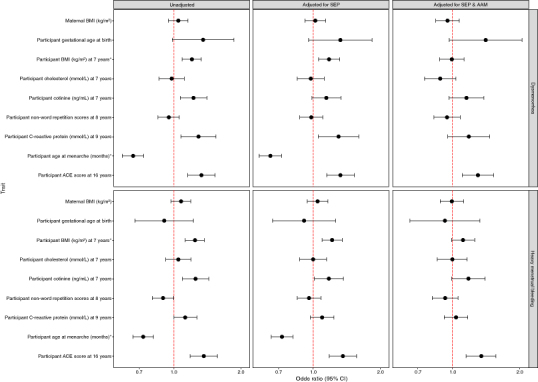
Coefficient plot representing continuous phenotypes associated with being a dysmenorrhea or heavy menstrual bleeding case in the hypothesis testing phase. * Identified as an associated trait *a priori*.

We found that, compared with unexposed participants, participants who had been exposed to smoke prenatally and who had smoked or drunk alcohol by age 13 were more likely to report dysmenorrhea in the unadjusted models (Figure 3). Higher BMI, CRP, cotinine and ACE score was also associated with an increased likelihood of reporting dysmenorrhea (Figure 4). Following adjustment for SEP and AAM, these effects attenuated, except for smoking at 13 years and ACE score at 16 years (aOR 1.61 95% CI: 1.11–2.33 and aOR 1.30 95% CI: 1.11–1.53, respectively (Supplementary Table S1).

In the unadjusted HMB models, participants exposed to smoke and maternal HDP prenatally were more likely to report HMB during puberty; smoking and drinking alcohol by the age of 13 was also associated with an increased likelihood of reporting HMB (Figure 3). Higher BMI, cotinine and ACE score was positively associated with HMB in the unadjusted models (Figure 4). Following adjustment for SEP and AAM, these effects attenuated, except for smoking at 13 years and ACE score at 16 years (aOR 2.35 95% CI: 1.69–3.26 and aOR 1.35 95% CI: 1.15–1.57, respectively) (Supplementary Table S2).

Additionally, although all confidence intervals crossed the null, dysmenorrhea (but not HMB) was consistently associated with higher gestational age at delivery with relatively large effect estimates (aOR 1.41 95% CI: 0.97–2.06) (Figure 4).

### 3.3. Sensitivity analysis

In the sensitivity analysis, where we ran the same EWAS analysis for each symptom with cases of thyroid problems at 17 years and PCOS or endometriosis at 22 years removed, four hits from the primary analysis persisted for dysmenorrhea (Supplementary Table S3) and one hit persisted for HMB (Supplementary Table S3). The effect estimates in the sensitivity analysis all followed the same direction as the estimates from the primary analysis, and on the most part strengthened, except for cg04737758 which attenuated toward the null. We then ran the hypothesis-testing phase in the cohort with these cases removed. The direction of the associations with all traits didn't change and the conclusions made from the primary analysis didn't change (Supplementary Figures S3 & S4).

When we performed the hypothesis-testing phase in less severe cases of each symptom, most associations attenuated toward the null. Associations that were stronger in the sensitivity analysis of less severe cases (exposure to maternal pre-eclampsia and cotinine at age 7 for HMB and gestational age at birth for dysmenorrhea) were in the same direction as the primary analysis (Supplementary Figures S5 & S6).

Of the dysmenorrhea cases, <5 participants reported pain for the first time after their methylation measurement was taken. For HMB, <5 reported prior to methylation measurement. When these were excluded from the hypothesis-testing phase, our conclusions did not change (Supplementary Figures S7 & S8).

## 4. Discussion

In this study, we corroborated previously identified associations (i.e., BMI and smoking) and generated new hypotheses about phenotypes (i.e., ACEs and alcohol consumption) that may contribute to the development of adolescent dysmenorrhea and HMB. These hypotheses warrant further investigation in a causally motivated framework, as what we present here is purely associational. We were able to replicate previously reported associations with own smoking [12,47] and higher BMI [15,48] with both menstrual symptoms, guided by methylation markers which supports its utility as a hypothesis-generating approach. We believe that this is the first study to present evidence that that early life experiences such as ACEs and prenatal exposures such as maternal smoking are associated with these conditions. The identification of both previously identified associations, as well as novel condition-phenotype relationships, suggests that the use of a hypothesis-generating EWAS approach may be useful to identify associations for future causal inference work.

### 4.1. Previous literature

EWAS investigate differentially CpGs in relation to a single phenotype and generates hypotheses, as the associations they identify between a trait of interest and differentially methylated CpGs may either be causal (i.e., the CpG/gene causes the trait), represent a historical exposure (i.e., flags someone as a smoker, for example), or highlight confounding. All these potential explanations for associations are useful when thinking about disease etiology and future analyses [4]. Their findings can focus subsequent causal analyses and can be implemented in scenarios where epigenetic data, as well as rich phenotypic data, are available. Genome-wide association studies (GWAS) are useful in such scenarios but only focus on genetics, whereas EWAS allows us to leverage confounding by incorporating exposures throughout the early life that might be associated with later life conditions. In the context of exposome-wide association studies (ExWAS), EWAS has been employed to reduce exposome dimension and make efficiency gains [49], reflecting the intention in this present study but here, to improve efficiency in subsequent observational, non-WAS analyses.

In order to test the combination of minimally adjusted EWAS with the hypothesis-testing phase for identifying associations, we wanted to use example conditions where few associations have been previously confirmed, so that there would be scope to identify novel traits. ALSPAC has repeat measures of the presence of dysmenorrhea and HMB throughout adolescence. In the literature, known causes and risk factors (outside diagnosed gynecological problems) are scant, with some evidence suggesting smoking [47] and BMI [15,48] are associated with these conditions.

### 4.2. Novel findings

Having performed two EWAS, one for dysmenorrhea and the other for HMB, we identified seven and two differentially methylated CpG sites, respectively. The seven dysmenorrhea CpG hits and their resident gene regions were associated with 31 individual traits, including negative associations with smoking (previously identified) and alcohol consumption, child abuse and pre-eclampsia (novel). The two HMB CpG hits and their resident gene regions were associated with ten individual traits including smoking (previously identified) and gestational age, total cholesterol and pre-eclampsia (novel).

In the hypothesis-testing phase, we identified that smoking and alcohol consumption at age 13, as well as ACE score at age 16 were all associated with dysmenorrhea and HMB, including after adjustments for SEP and AAM. Although causal effects cannot be inferred from these analyses, they provide evidence that further investigation into these traits may be able to illuminate mechanisms by which they are associated with dysmenorrhea and HMB.

### 4.3. Strengths & limitations

We present a potentially useful, epigenetic-based approach that can be implemented by leveraging confounding to identify phenotype-phenotype associations even on a small scale, provided there is sufficient access to epigenetic and phenotypic data. The small number of cases and controls limit our concerns that our findings may be a result of multiple testing. Given that the hypothesis-testing phase relies on a minimally adjusted EWAS analysis, a full complement of confounders is not required, thus participants are less frequently excluded for not having complete covariate data, which is particularly useful if case and control numbers are small. The G1 puberty questionnaires in ALSPAC were sent out multiple times allowing us several timepoints across adolescence within which to identify cases for our conditions. Despite cord blood methylation being available in ALSPAC, we chose to use adolescent DNA methylation because we wanted to identify exposures across the life course to date that may be associated with the development of these conditions, additionally to genotypic differences.

A major limitation in this study was power, which was low particularly compared with other EWAS. However, prior work leveraging small number of participants in epigenetic analyses has driven hypotheses in other fields; for example, a small hypothesis-generating EWAS of paternal smoking identified offspring DNA methylation that might be associated with development [50] that was carried forward by another group investigating drivers of childhood autism [51]. Sample size was also a problem in the hypothesis-testing phase, as some phenotypes were underpowered in ALSPAC. This further reinforces the need for causally motivated analyses in other cohorts.

It is likely that larger genes may have been over-represented by the approach we took to identify phenotypes given that we investigated phenotypes associated with both the differentially methylated CpG and its resident gene. We chose to accept this limitation on the basis that it widened the net we were casting for generated hypotheses. Our approach relies on previous research that is published in the EWAS Catalog. Therefore, it is only able to identify associations with phenotypes where some epigenetic research has been previously conducted. Additionally, the EWAS Catalog is biased toward heavily investigated phenotypes, such as smoking, so such phenotypes were more likely to be identified in the hypothesis-generating phase than less frequently investigated phenotypes. In line with our hypothesis-generating approach, we opted to extract *all* EWAS Catalog phenotypes associated with the genes we identified in our menstrual EWAS. However, if future studies are concerned about biases in the EWAS Catalog, an enrichment analysis (e.g., Fisher's Exact Test) could be used to identify those phenotypes that have an unusually large representation in the list of phenotypes associated with the candidate CpGs identified in the discovery EWAS, relative to their representation in the entire Catalog. More broadly, most EWAS, including our own, are biased by the coverage of genes on the arrays used to obtain epigenetic data.

Misclassification of case status was a concern, given the mix of caregiver and adolescent responses used to ascertain cases of each symptom; however, there was minimal disagreement between answers given at multiple timepoints. Although multiple testing burden was high (∼470K probes) compared with other hypothesis-generating approaches, like phenome-wide wide association studies (PheWAS), data are not appropriately processed in ALSPAC for PheWAS. Thus, we propose the use of our approach where data are not coded up for PheWAS in the presence of epigenetic and phenotypic data. Temporality was well established in the hypothesis-generating phase, whereby the majority of those defined as a case in the EWAS reported the first instance of their symptom prior to their methylation being measured, however this was not as simple in the following phase for phenotypes and symptoms. Despite our best efforts to maintain sensible temporality in the hypothesis-testing phase, sometimes we were not able to derive phenotype variables (exposure) that were definitively before the condition onset (outcome). For example, the ACE score variable used to explore the potential association between child abuse and the worked example conditions was a composite variable of ACEs up to the age of 16, given as a score [46]. It is likely that some of the participants in the hypothesis-testing phase analysis of ACE score may have had ACEs that contributed to their score at age 15 or 16, where the onset of their dysmenorrhea was earlier in puberty. However, as we weren't doing any causal analyses, merely attempting to identify associations between potential risk factors and menstrual conditions, temporality wasn't of utmost importance to uphold.

The findings from our hypothesis-testing phase of dysmenorrhea and HMB were only internally validated in the wider ALSPAC cohort, as opposed to in another cohort; replication is crucial to draw further inference from these tentative findings. It is important that future studies investigating the potential associations identified here do so in other independent cohorts where menstrual health data are available. It is likely that some characteristics explored in the hypothesis-testing phase are highly correlated; future studies where temporality of menstrual symptoms and potential risk factors is well-established would be best-placed to investigate these in more detail. The associations we observe in the hypothesis-testing phase may be completely mediated or modified by the much higher prevalence of hormonal contraception use in those with either condition; it is important to investigate the role of contraception for each association separately in future analyses. As Sawyer et al. point out, conditioning on hormonal contraception use might introduce collider bias if our phenotype of interest and condition are likely to influence contraception use [24]. Finally, we are aware that the definition for severity for each condition (those who had visited a doctor for the symptom) may instead reflect socioeconomic, cultural and personal factors for certain participants that will have influenced why they sought medical advice for their symptoms while others did not, which has been highlighted in other fields [52].

## 5. Conclusion

We used an epigenome-led approach to generate hypotheses regarding potential risk factors, using dysmenorrhea and HMB as example phenotypes. The novel approach used here, leveraging both a hypothesis-generating and -testing phase, as well as confounding relationships, detected both known and novel associations between menstrual symptoms and environmental or physiological exposures. This novel approach could be added to the arsenal of exploratory analyses that drive hypotheses for future causal analyses in a range of understudied health problems, including menstrual health epidemiology.

## Supplementary Material

Supplementary Figures S1-S8 and Tables S1-S3

## Data Availability

Access to ALSPAC data is through a system of managed open access (http://www.bristol.ac.uk/alspac/researchers/access/). Scripts used to clean and analyse the data for this study can be found on GitHub (https://github.com/flozoemartin/MP1/).
