# Supplementary material for: A novel hypothesis-generating approach for detecting phenotypic associations using epigenetic data
Source: Epigenomics. 2024 Jul 17;16(11-12):851–64. doi: 10.1080/17501911.2024.2366157 (PMC11370959; doi:10.1080/17501911.2024.2366157)
Supplement: Supplementary Figures S1-S8 and Tables S1-S3 [file IEPI_A_2366157_SM0001.zip › Supplementary material.docx]

Supplementary material

**Table S1** Comparison of dysmenorrhea cases and controls for each binary and continuous characteristic (as shown in Figure 3) where G0 refers to a maternal characteristic and G1 refers to enrolled adolescent characteristic

|  | **OR**  **(95% CI)** | ***p*-value** | **aOR**  **(SEP only)**  **(95% CI)** | ***p*-value** | **aOR**  **(AAM & SEP)** | **(95% CI)** |
| --- | --- | --- | --- | --- | --- | --- |
| **Binary characteristics** | | | | | | |
| **Prenatal** | | | | | | |
| G0 university degree* | 0.61  (0.46 to 0.82) | 1.02$\times{10}^{-3}$ | - | - | - | - |
| G0 alcohol consumption during pregnancy | 1.10  (0.84 to 1.45) | 0.476 | 1.16  (0.88 to 1.52) | 0.303 | 1.14  (0.83 to 1.57) | 0.403 |
| G0 smoking during pregnancy | 1.34  (1.05 to 1.71) | 1.69$\times{10}^{-2}$ | 1.22  (0.94 to 1.59) | 0.129 | 1.09  (0.81 to 1.48) | 0.571 |
| Any G0 HDP | 1.15  (0.89 to 1.48) | 0.297 | 1.15  (0.89 to 1.50) | 0.290 | 1.02  (0.75 to 1.38) | 0.919 |
| G0 preeclampsia | 0.68  (0.32 to 1.47) | 0.329 | 0.73  (0.34 to 1.59) | 0.428 | 0.66  (0.28 to 1.60) | 0.362 |
| **Age 13 years** | | | | | | |
| G1 has drunk alcohol before | 1.54  (1.18 to 2.02) | 1.67$\times{10}^{-3}$ | 1.51  (1.13 to 2.00) | 4.64$\times{10}^{-3}$ | 1.37  (0.99 to 1.89) | 5.61$\times{10}^{-2}$ |
| G1 has smoked cigarettes before* | 2.19  (1.61 to 2.99) | 6.25$\times{10}^{-7}$ | 2.23  (1.60 to 3.12) | 2.34$\times{10}^{-6}$ | 1.61  (1.11 to 2.33) | 1.23$\times{10}^{-2}$ |
| **End of puberty** | | | | | | |
| G1 has used oral contraception* | 8.30  (6.70 to 10.3) | 3.13$\times{10}^{-82}$ | 7.76  (6.18 to 9.76) | 3.81$\times{10}^{-69}$ | 7.55  (5.80 to 9.82) | 2.34$\times{10}^{-51}$ |
| G1 has reported a comorbidity (thyroid problems, PCOS, endometriosis)* | 2.11  (1.27 to 3.48) | 3.70$\times{10}^{-5}$ | 1.60  (0.93 to 2.76) | 0.089 | 1.76  (0.92 to 3.37) | 0.086 |
| **Continuous characteristics** | | | | | | |
| **Prenatal** | | | | | | |
| G0 body mass index (BMI) during pregnancy, kg/m^2^ | 1.05  (0.94 to 1.16) | 0.390 | 1.02  (0.92 to 1.14) | 0.682 | 0.95  (0.84 to 1.07) | 0.421 |
| **Delivery** | | | | | | |
| G1 gestational age at delivery, weeks | 1.35  (0.98 to 1.86) | 0.065 | 1.32  (0.95 to 1.84) | 0.094 | 1.41  (0.97 to 2.06) | 0.074 |
| **Age 7 years** | | | | | | |
| G1 BMI, kg/m^2^ | 1.20  (1.09 to 1.33) | 2.05$\times{10}^{-4}$ | 1.18  (1.06 to 1.31) | 2.82$\times{10}^{-3}$ | 0.99  (0.88 to 1.13) | 0.92 |
| G1 cholesterol, mmol/L | 0.98  (0.86 to 1.11) | 0.72 | 0.97  (0.85 to 1.12) | 0.72 | 0.88  (0.75 to 1.03) | 0.12 |
| G1 cotinine, ng/mL | 1.22  (1.07 to 1.41) | 3.93$\times{10}^{-3}$ | 1.15  (0.99 to 1.33) | 7.43$\times{10}^{-2}$ | 1.16  (0.97 to 1.38) | 0.11 |
| **Age 8 years** | | | | | | |
| G1 non-word repetition | 0.95  (0.85 to 1.06) | 0.34 | 0.98  (0.87 to 1.11) | 0.75 | 0.95  (0.83 to 1.08) | 0.42 |
| **Age 9 years** | | | | | | |
| G1 C-reactive protein (CRP), mmol/L | 1.29  (1.07 to 1.54) | 6.09$\times{10}^{-3}$ | 1.30  (1.06 to 1.60) | 1.24$\times{10}^{-2}$ | 1.18  (0.95 to 1.47) | 0.13 |
| **Teen** | | | | | | |
| G1 age at menarche, months* | 0.66  (0.59 to 0.73) | 4.60$\times{10}^{-14}$ | 0.64  (0.57 to 0.72) | 1.02$\times{10}^{-13}$ | - | - |
| **Age 16 years** | | | | | | |
| G1 adverse childhood experience (ACE) score | 1.33  (1.15 to 1.53) | 8.52$\times{10}^{-5}$ | 1.33  (1.15 to 1.53) | 1.08$\times{10}^{-4}$ | 1.30  (1.11 to 1.53) | 1.17$\times{10}^{-3}$ |

*Identified characteristic *a priori*.

**Table S2** Comparison of HMB cases and controls for each binary and continuous characteristic (as shown in Figure 4)

|  | **OR**  **(95% CI)** | ***p*-value** | **aOR**  **(SEP only)**  **(95% CI)** | ***p*-value** | **aOR**  **(AAM & SEP)** | **(95% CI)** |
| --- | --- | --- | --- | --- | --- | --- |
| **Binary characteristics** | | | | | | |
| **Prenatal** | | | | | | |
| Maternal university degree* | 0.47  (0.34 to 0.65) | 5.38$\times{10}^{-6}$ | - | - | - | - |
| Alcohol exposure during pregnancy | 1.03  (0.78 to 1.34) | 0.857 | 1.10  (0.84 to 1.45) | 0.487 | 1.12  (0.82 to 1.52) | 0.472 |
| Smoke exposure during pregnancy | 1.69  (1.33 to 2.13) | 1.20$\times{10}^{-5}$ | 1.50  (1.17 to 1.93) | 1.47$\times{10}^{-3}$ | 1.34  (1.01 to 1.79) | 4.32$\times{10}^{-2}$ |
| Any HDP | 1.34  (1.04 to 1.73) | 2.27$\times{10}^{-2}$ | 1.35  (1.04 to 1.75) | 2.61$\times{10}^{-2}$ | 1.19  (0.89 to 1.61) | 0.242 |
| Preeclampsia | 1.07  (0.55 to 2.09) | 0.848 | 1.10  (0.56 to 2.17) | 0.784 | 1.03  (0.48 to 2.20) | 0.928 |
| **Age 13 years** | | | | | | |
| Have drunk alcohol before | 1.36  (1.05 to 1.77) | 2.16$\times{10}^{-2}$ | 1.37  (1.04 to 1.80) | $2.74\times{10}^{-2}$ | 1.35  (1.00 to 1.82) | 4.67$\times{10}^{-2}$ |
| Have smoked cigarettes before* | 2.56  (1.93 to 3.14) | 1.05$\times{10}^{-10}$ | 2.75  (2.03 to 3.73) | $7.56\times{10}^{-11}$ | 2.35  (1.69 to 3.26) | 3.52$\times{10}^{-7}$ |
| **End of puberty** | | | | | | |
| Oral contraception use* | 8.82  (7.08 to 11.0) | 4.90$\times{10}^{-84}$ | 9.19  (7.24 to 11.7) | 2.55$\times{10}^{-74}$ | 9.77  (7.46 to 12.8) | 9.28$\times{10}^{-62}$ |
| Comorbidity (thyroid problems, PCOS, endometriosis) reported* | 2.39  (1.53 to 3.74) | 1.22$\times{10}^{-4}$ | 2.66  (1.64 to 4.31) | 7.39$\times{10}^{-6}$ | 2.71  (1.56 to 4.70) | 3.88$\times{10}^{-5}$ |
| **Continuous characteristics** | | | | | | |
| **Prenatal** | | | | | | |
| Maternal BMI, kg/m^2^ | 1.08  (0.97 to 1.19) | 0.164 | 1.05  (0.94 to 1.17) | 0.391 | 0.99  (0.88 to 1.12) | 0.922 |
| **Delivery** | | | | | | |
| Gestational age, weeks | 0.91  (0.67 to 1.22) | 0.520 | 0.91  (0.66 to 1.26) | 0.575 | 0.92  (0.64 to 1.33) | 0.671 |
| **Age 7 years** | | | | | | |
| BMI, kg/m^2^ | 1.24  (1.13 to 1.37) | 1.52$\times{10}^{-5}$ | 1.22  (1.09 to 1.35) | 2.68$\times{10}^{-4}$ | 1.12  (0.99 to 1.26) | 7.43$\times{10}^{-2}$ |
| Cholesterol, mmol/L | 1.05  (0.92 to 1.19) | 0.49 | 1.00  (0.87 to 1.15) | 0.99 | 1.00  (0.86 to 1.16) | 0.98 |
| Cotinine, ng/mL | 1.25  (1.09 to 1.43) | 1.09$\times{10}^{-3}$ | 1.18  (1.01 to 1.36) | 3.37$\times{10}^{-2}$ | 1.18  (1.00 to 1.40) | 5.70$\times{10}^{-2}$ |
| **Age 8 years** | | | | | | |
| Non-word repetition | 0.90  (0.80 to 1.00) | 5.18$\times{10}^{-2}$ | 0.96  (0.85 to 1.08) | 0.50 | 0.93  (0.81 to 1.06) | 0.27 |
| **Age 9 years** | | | | | | |
| CRP, mmol/L | 1.12  (1.00 to 1.27) | 5.45$\times{10}^{-2}$ | 1.10  (0.97 to 1.24) | 0.13 | 1.04  (0.92 to 1.17) | 0.54 |
| **Puberty** | | | | | | |
| Age at menarche, months* | 0.73  (0.66 to 0.81) | 4.17$\times{10}^{-9}$ | 0.73  (0.65 to 0.81) | 2.08$\times{10}^{-8}$ | - | - |
| **Age 16 years** | | | | | | |
| ACE score | 1.36  (1.18 to 1.56) | 1.30$\times{10}^{-5}$ | 1.36  (1.18 to 1.57) | 2.09$\times{10}^{-5}$ | 1.35  (1.15 to 1.57) | 1.51$\times{10}^{-4}$ |

### Sensitivity analysis

**Table S3** Differentially methylated CpG sites in hypothesis-generating EWAS with comorbidity cases removed compared with the effect estimates from the primary EWAS (dysmenorrhea and HMB)

| **Probe ID** | $\boldsymbol{\beta}$  (ex. comorbidities)  (95%CI) | ***p*-value** | $\boldsymbol{\beta}$  (primary analysis)  (95%CI) | ***p*-value** | **Absolute % change** |
| --- | --- | --- | --- | --- | --- |
| **Dysmenorrhea** | | | | | |
| cg08142094 | -0.038  (-0.053 to -0.023) | 2.73${\times10}^{-6}$ | -0.036  (-0.050 to -0.022) | 1.02${\times10}^{-6}$ | +5% |
| cg23012731 | -0.009  (-0.013 to -0.006) | 5.12${\times10}^{-7}$ | -0.008  (-0.011 to -0.005) | 3.53${\times10}^{-6}$ | +12.5% |
| cg04737758 | 0.036  (0.018 to 0.054) | 8.97${\times10}^{-5}$ | 0.042  (0.025 to 0.058) | 1.13${\times10}^{-6}$ | -14.3% |
| cg15017604 | 0.030  (0.017 to 0.043) | 8.87${\times10}^{-6}$ | 0.026  (0.015 to 0.037) | 9.39${\times10}^{-6}$ | +15.4% |
| **Heavy menstrual bleeding** | | | | | |
| cg24196053 | -0.011  (-0.016 to -0.005) | 8.33${\times10}^{-5}$ | -0.010  (-0.014 to -0.006) | 1.50${\times10}^{-5}$ | +10% |

**
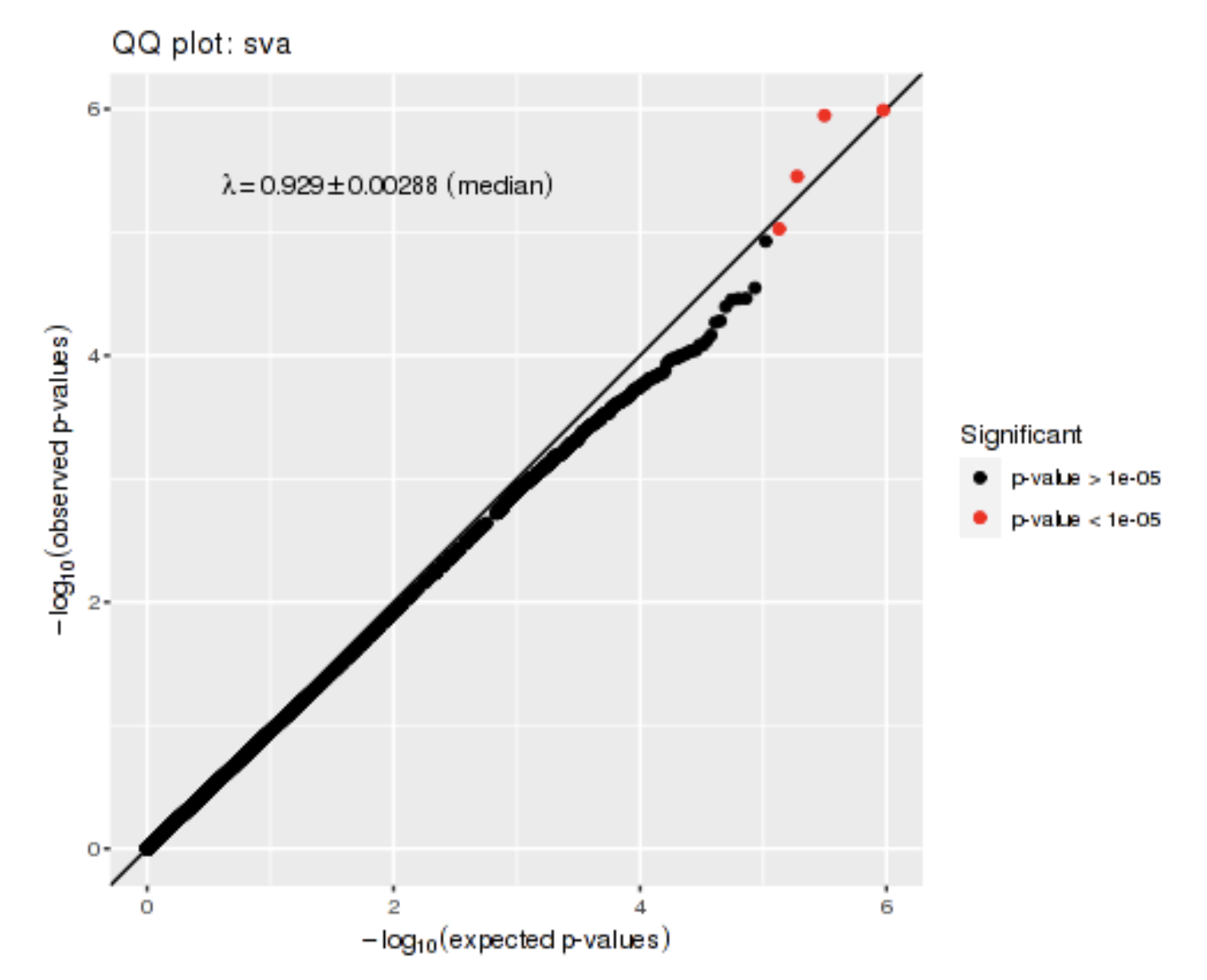
**

**Figure S1** QQ plot for dysmenorrhea EWAS.


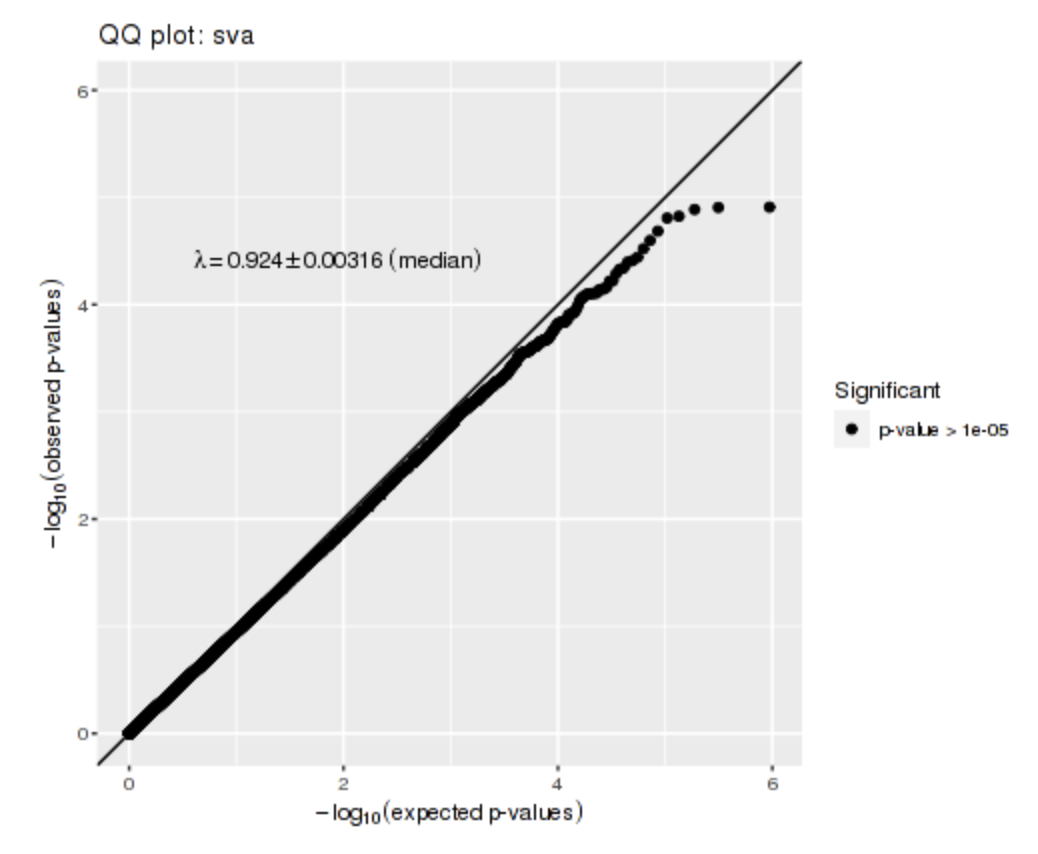


**Figure S2** QQ plot for HMB EWAS.


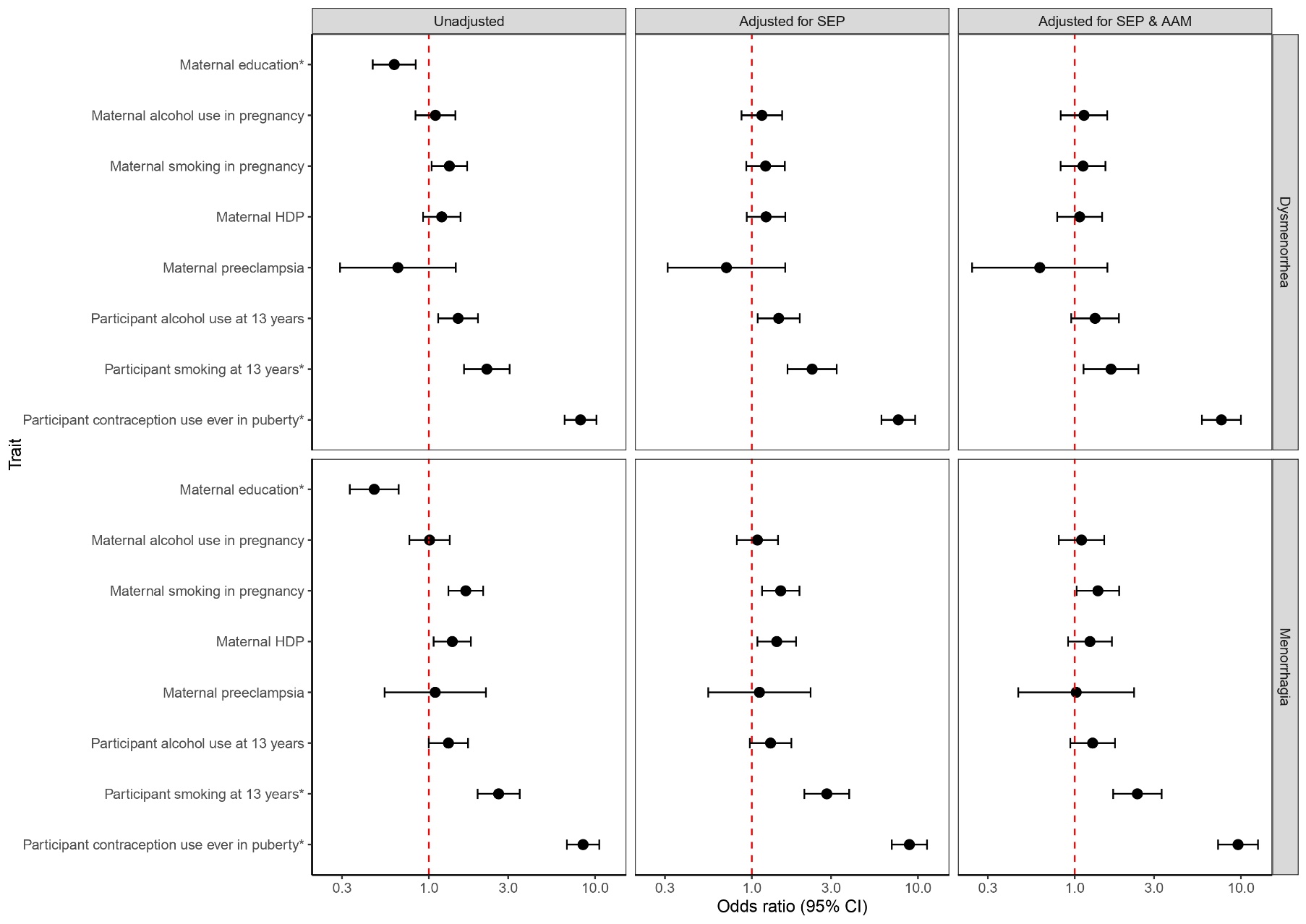


**Figure S3** Coefficient plot representing binary characteristics associated with dysmenorrhea and HMB with participants with a comorbidity removed

* Identified as an associated characteristic *a priori*

*
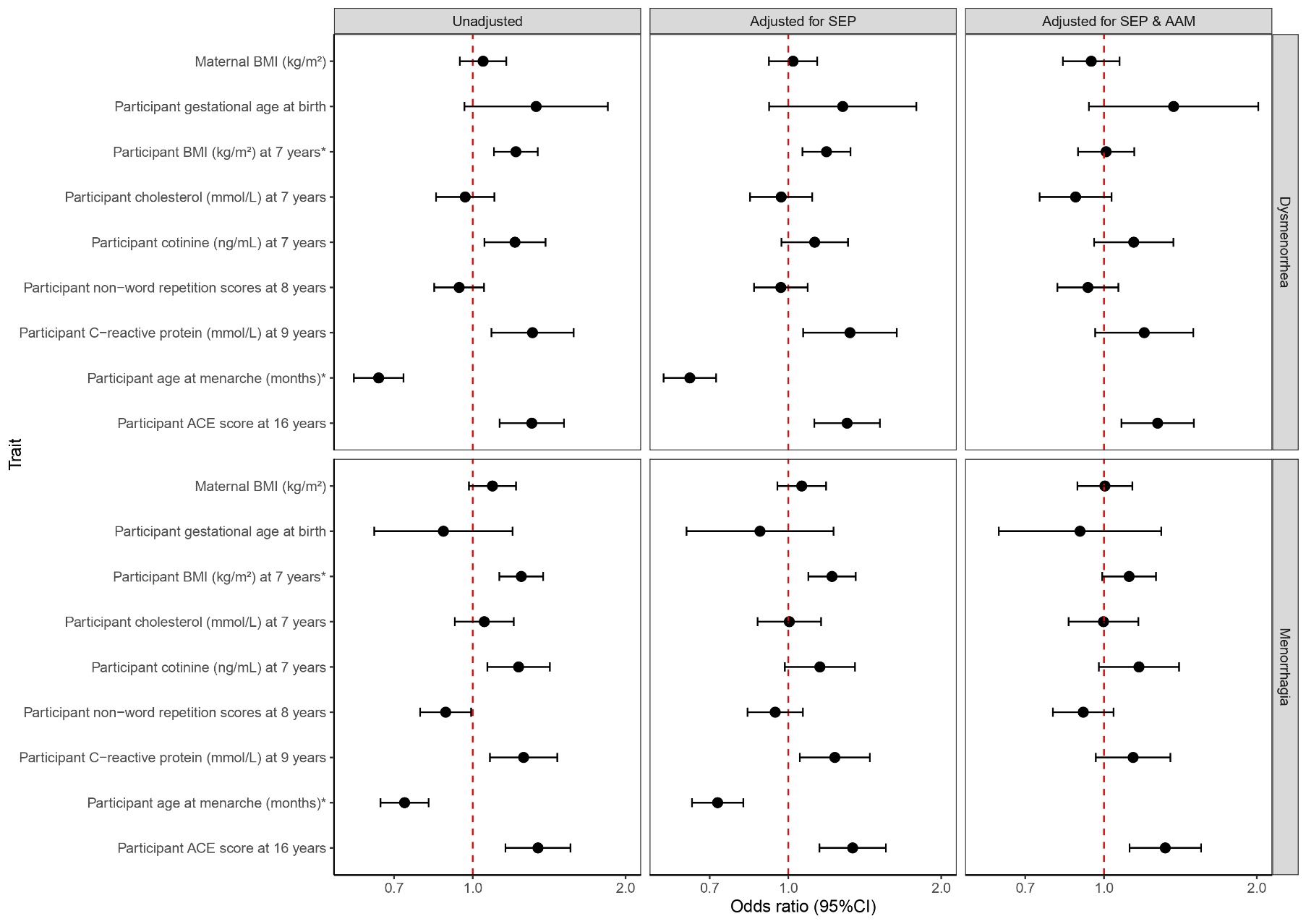
*

**Figure S4** Coefficient plot representing continuous characteristics associated with dysmenorrhea and HMB with participants with a comorbidity removed

* Identified as an associated characteristic *a priori*

*
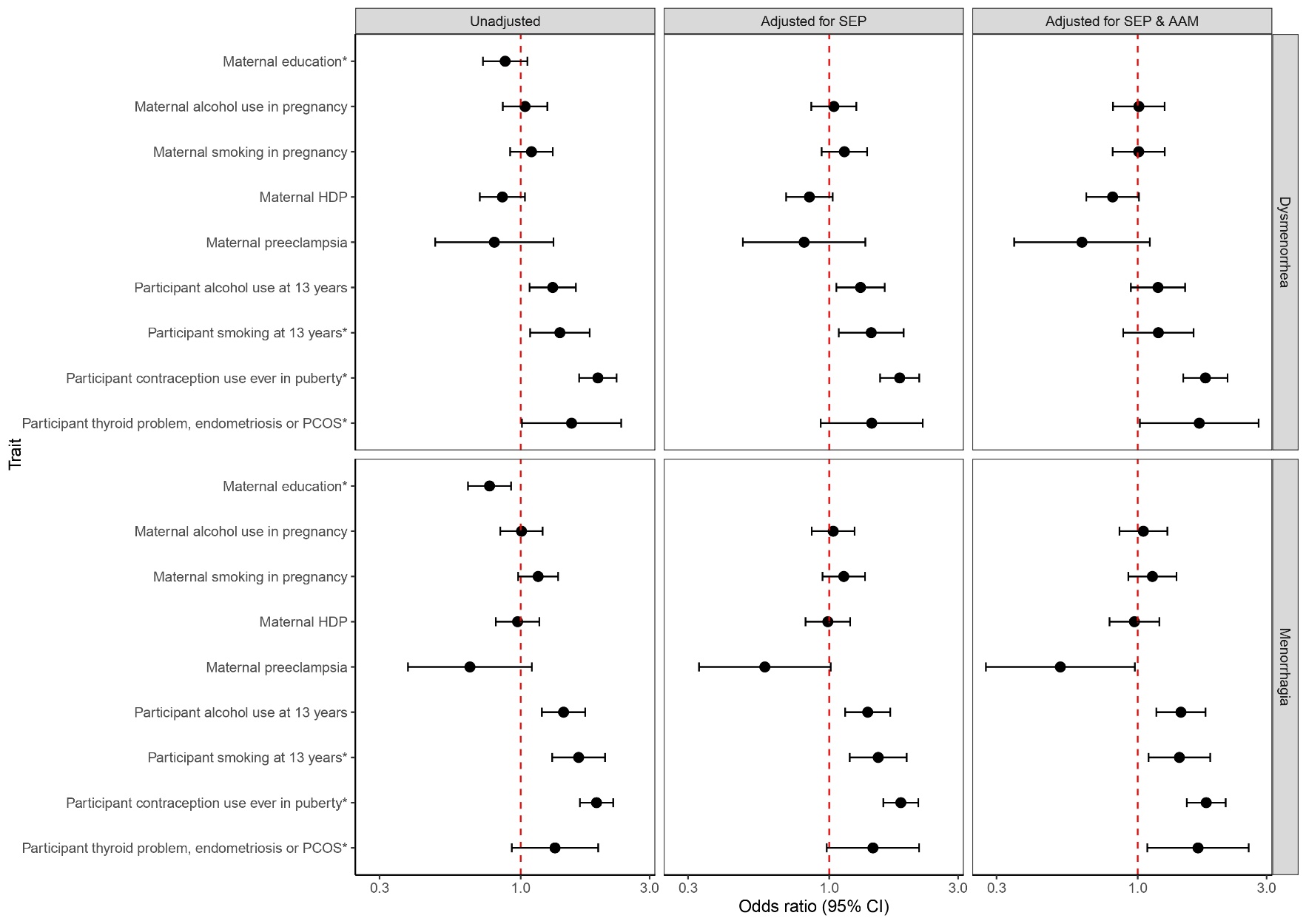
*

**Figure S5** Coefficient plot representing binary traits associated with less severe dysmenorrhea and HMB

* Identified as an associated trait *a priori*

*
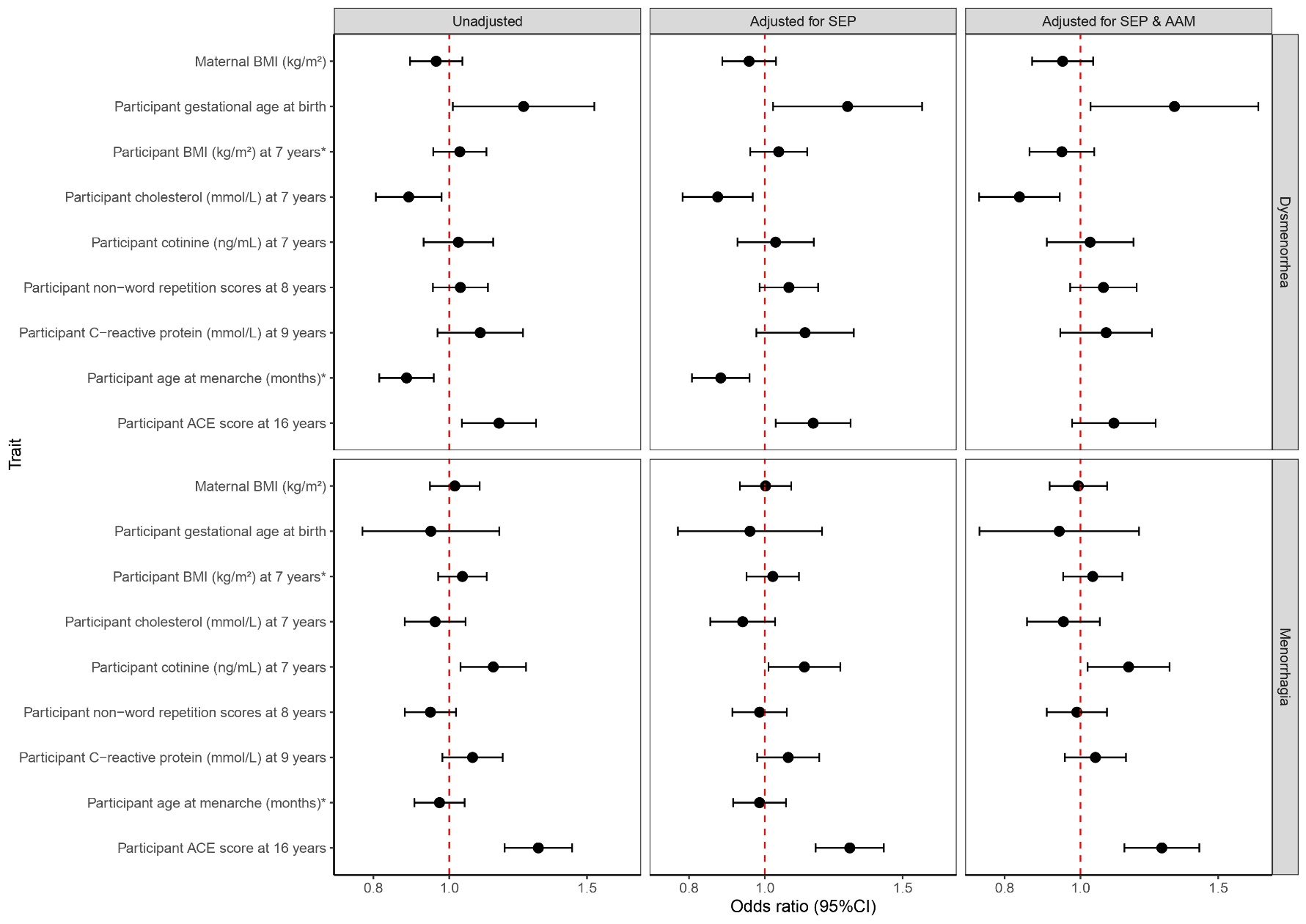
*

**Figure S6** Coefficient plot representing continuous traits associated with less severe dysmenorrhea and HMB

* Identified as an associated trait *a priori*

*
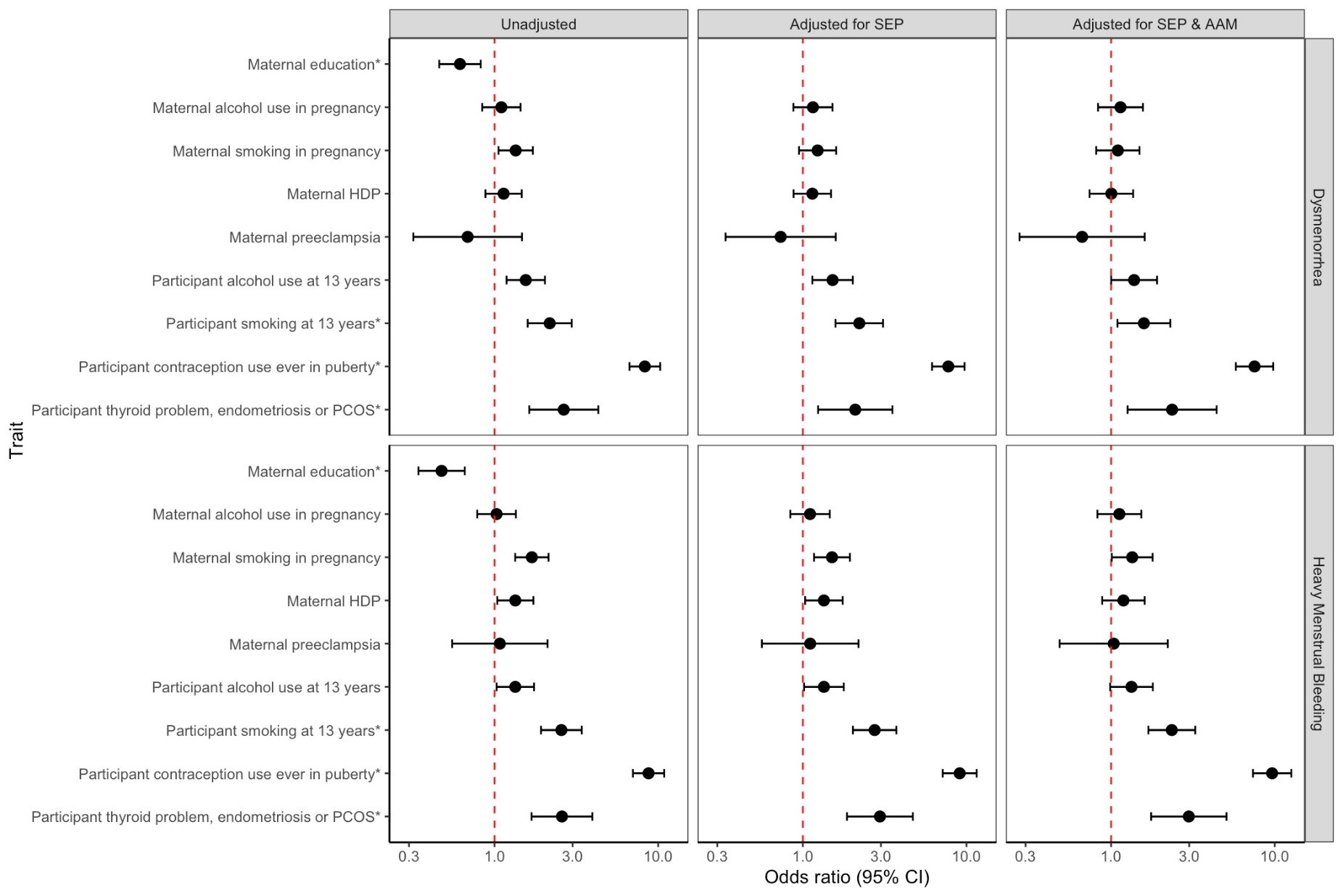
*

**Figure S7** Coefficient plot representing binary traits associated with those whose methylation was measured prior to their first report of either symptom removed

* Identified as an associated trait *a priori*


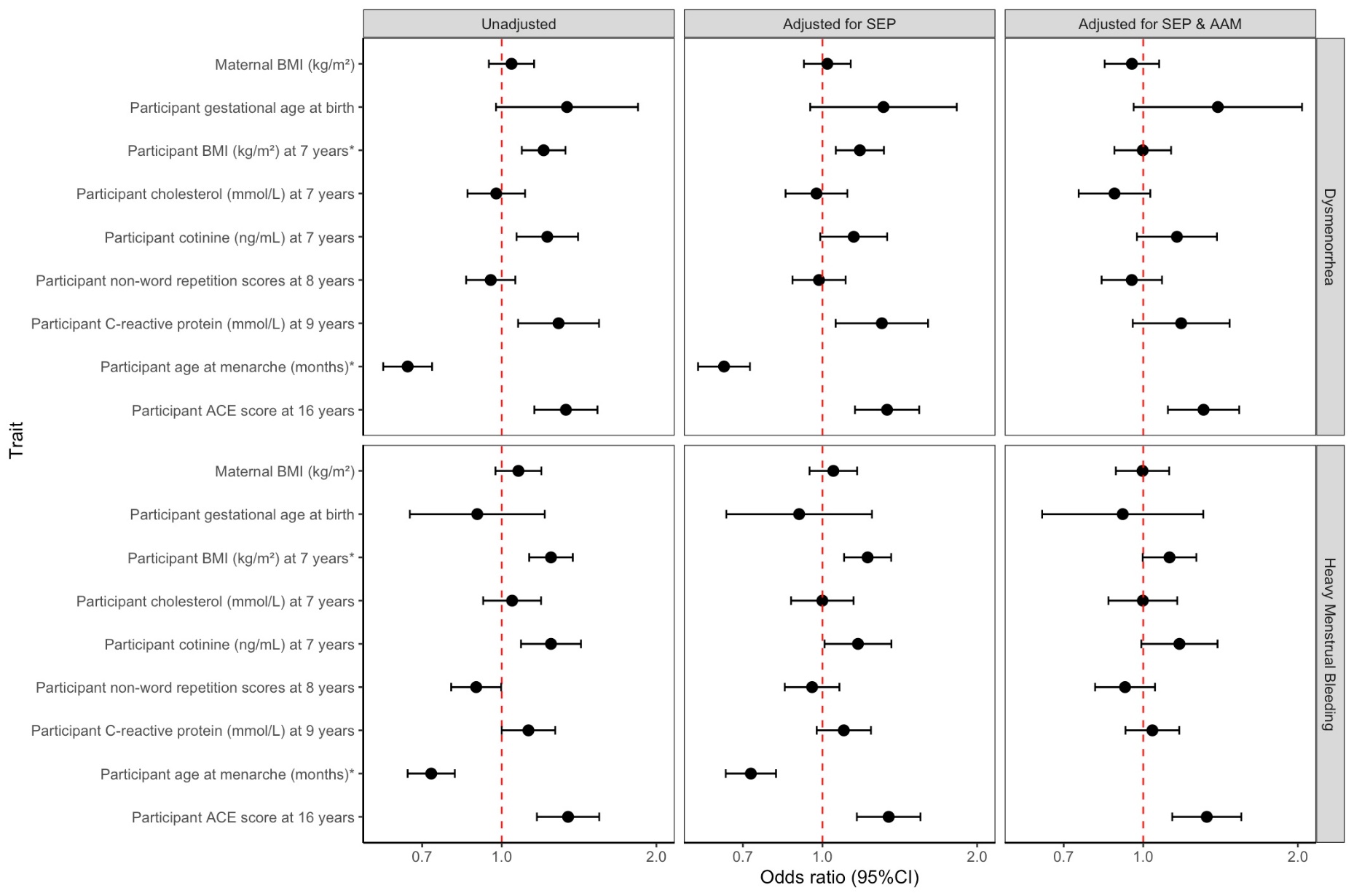


**Figure S8** Coefficient plot representing continuous traits associated with those whose methylation was measured prior to their first report of either symptom removed

* Identified as an associated trait *a priori*
