# Supplementary figures and images for: A novel hypothesis-generating approach for detecting phenotypic associations using epigenetic data
Source: Epigenomics. 2024 Jul 17;16(11-12):851–64. doi: 10.1080/17501911.2024.2366157 (PMC11370959; doi:10.1080/17501911.2024.2366157)

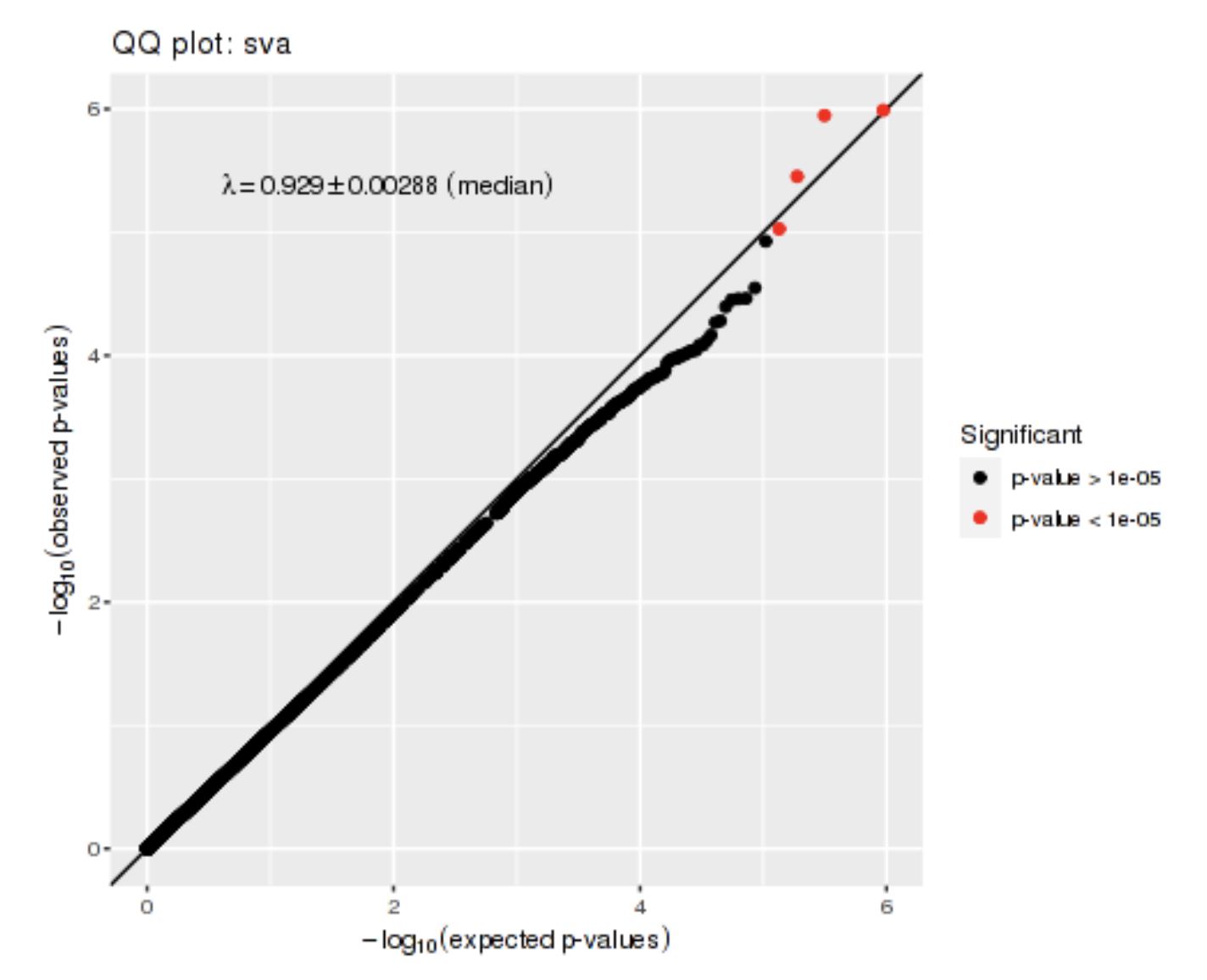

Supplement: Supplementary Figures S1-S8 and Tables S1-S3 [file IEPI_A_2366157_SM0001.zip › Figure S1.png]

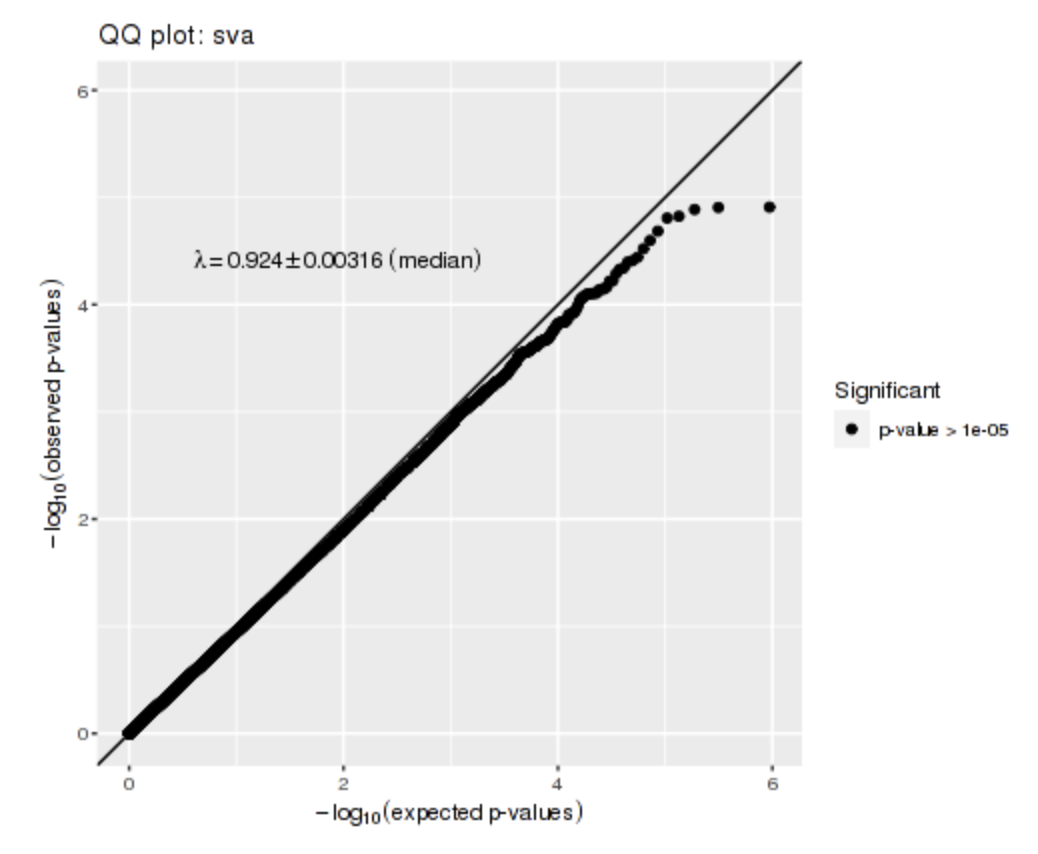

Supplement: Supplementary Figures S1-S8 and Tables S1-S3 [file IEPI_A_2366157_SM0001.zip › Figure S2.png]

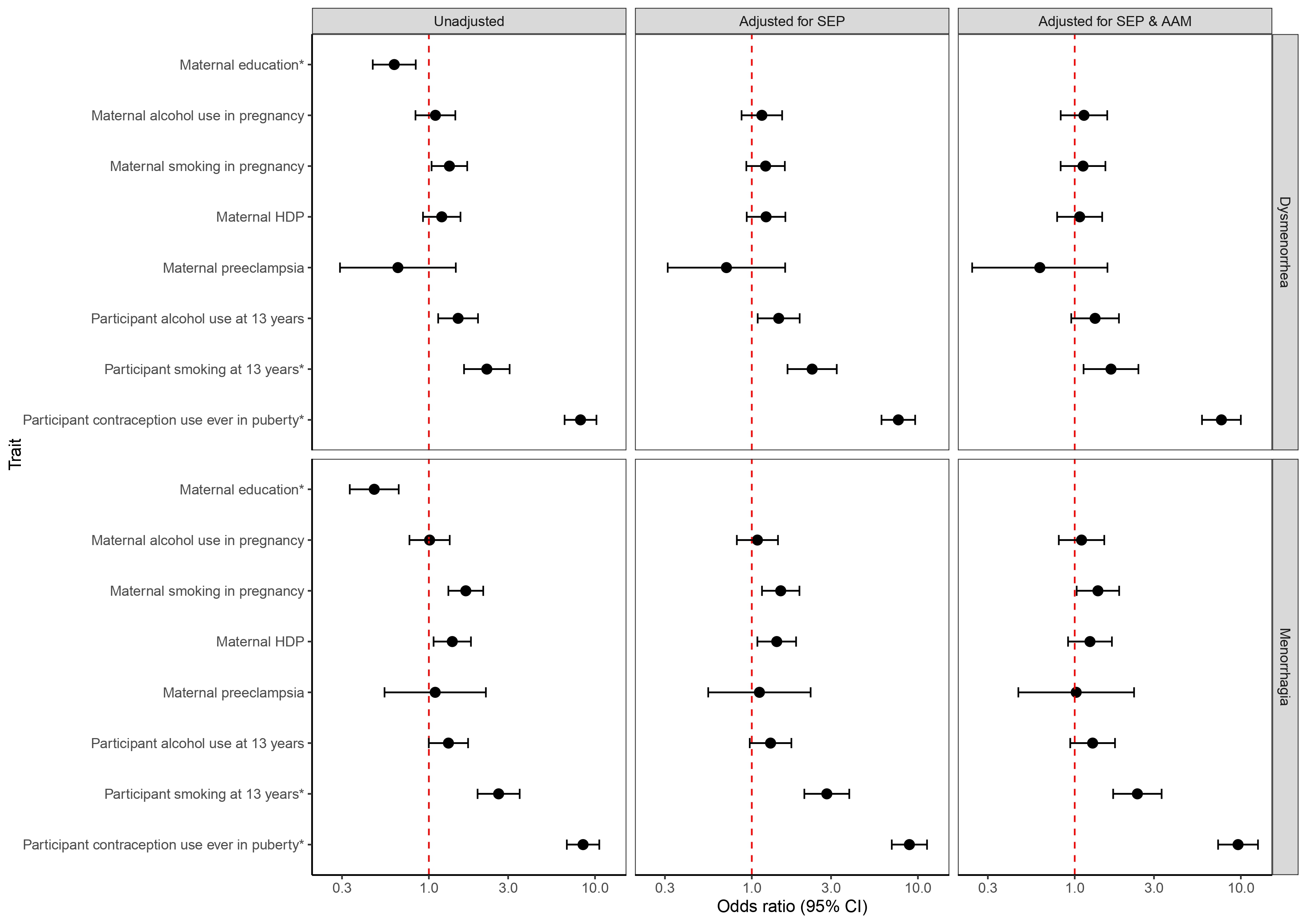

Supplement: Supplementary Figures S1-S8 and Tables S1-S3 [file IEPI_A_2366157_SM0001.zip › Figure S3.jpg]

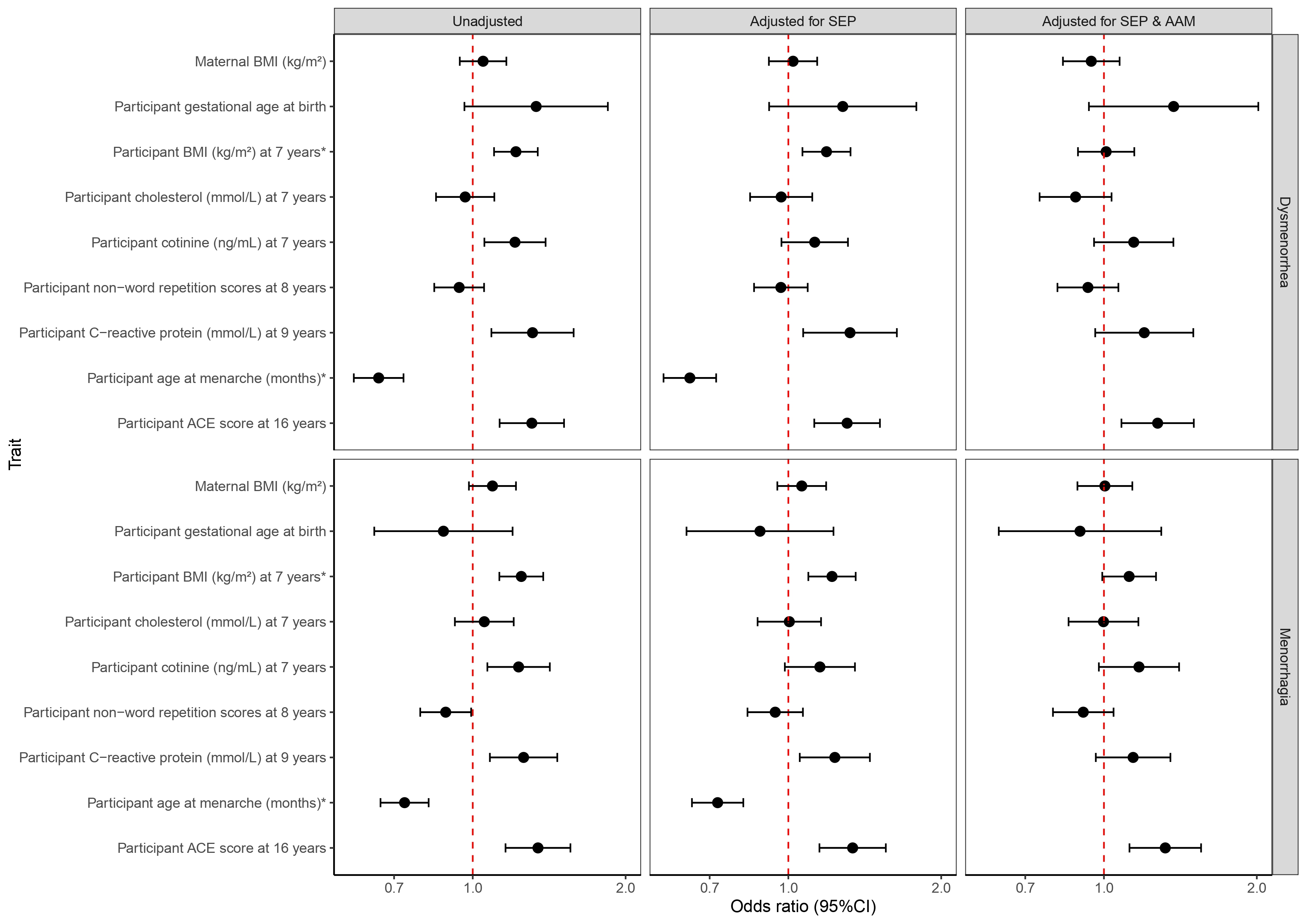

Supplement: Supplementary Figures S1-S8 and Tables S1-S3 [file IEPI_A_2366157_SM0001.zip › Figure S4.jpg]

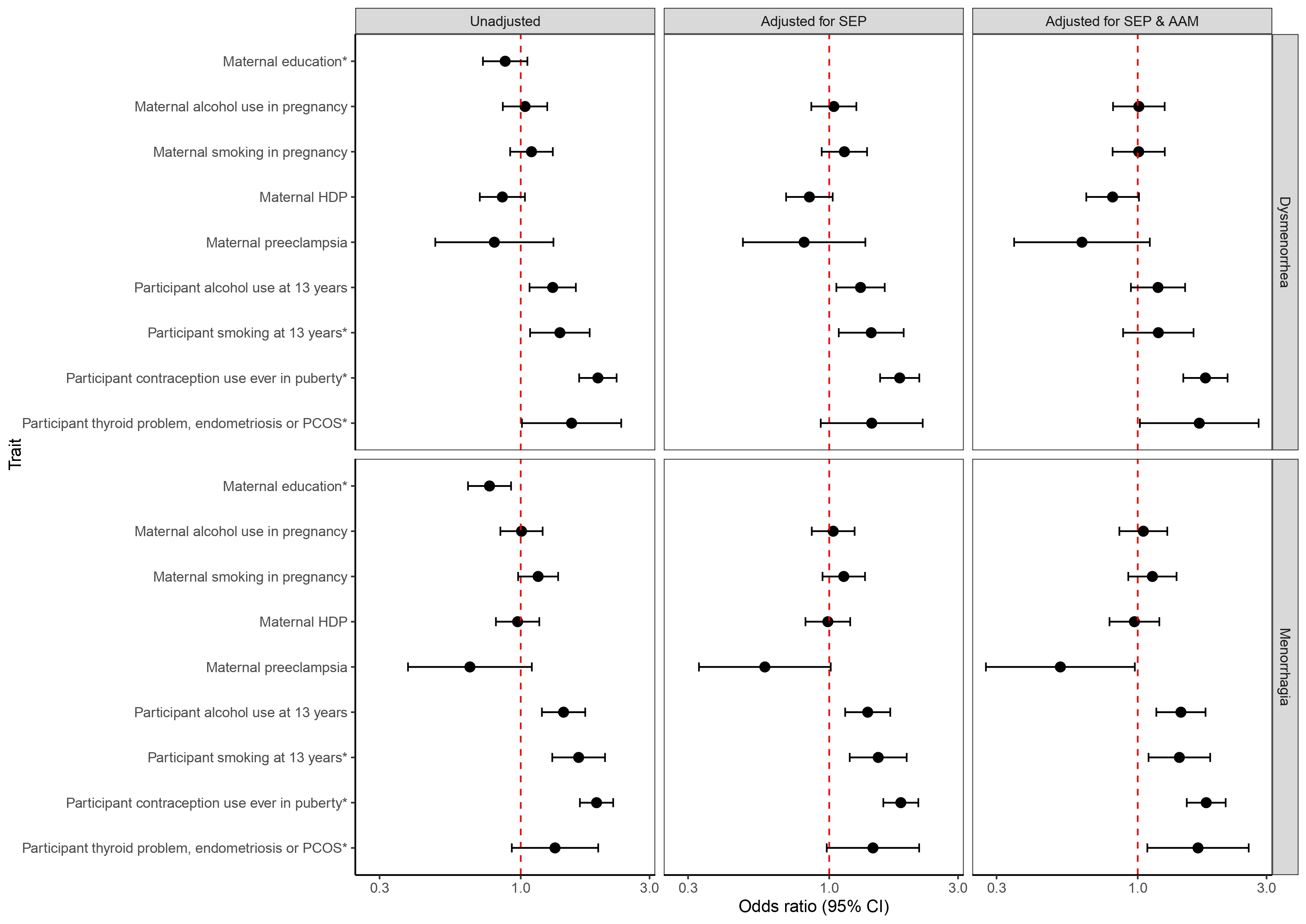

Supplement: Supplementary Figures S1-S8 and Tables S1-S3 [file IEPI_A_2366157_SM0001.zip › Figure S5.jpg]

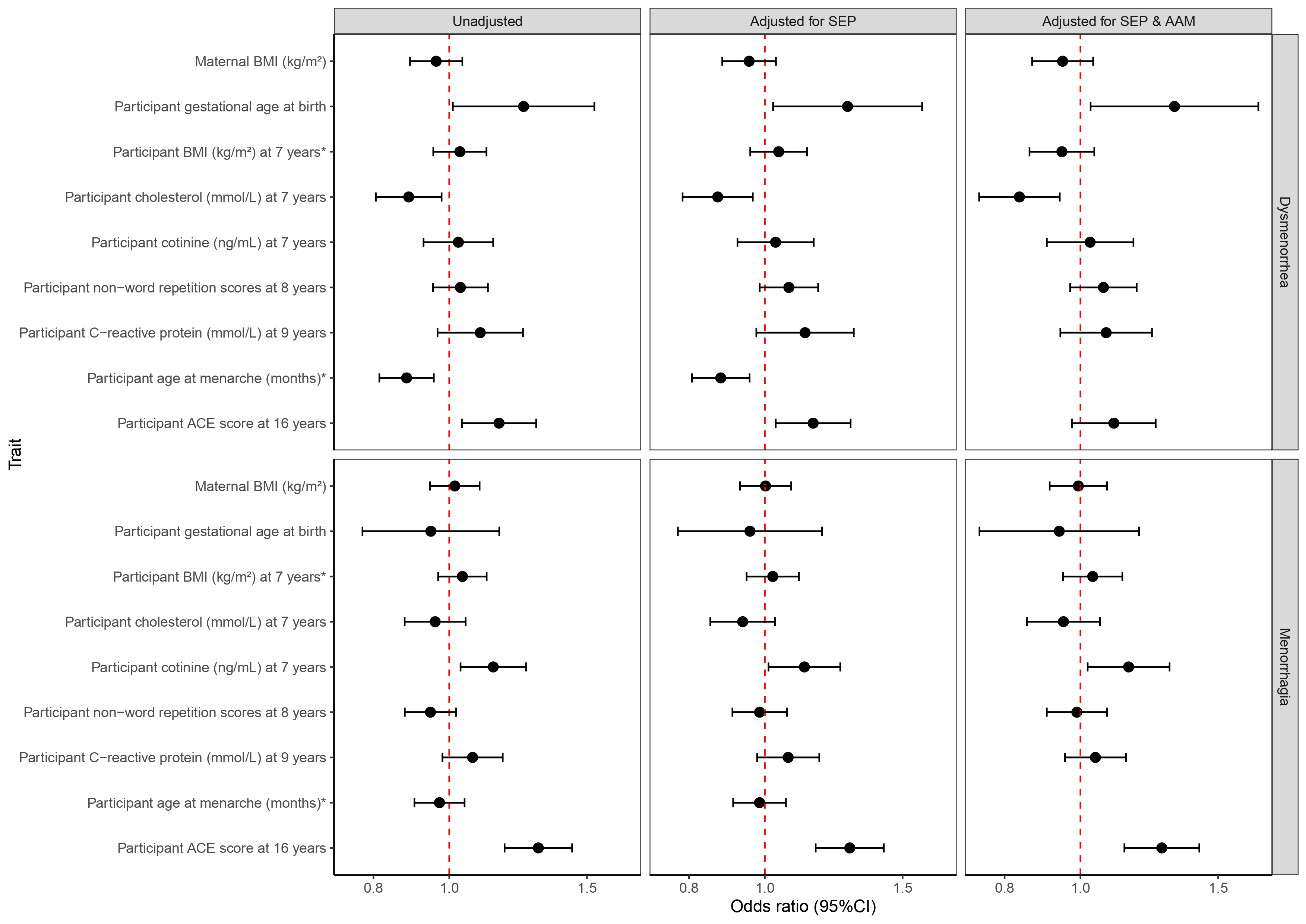

Supplement: Supplementary Figures S1-S8 and Tables S1-S3 [file IEPI_A_2366157_SM0001.zip › Figure S6.jpg]

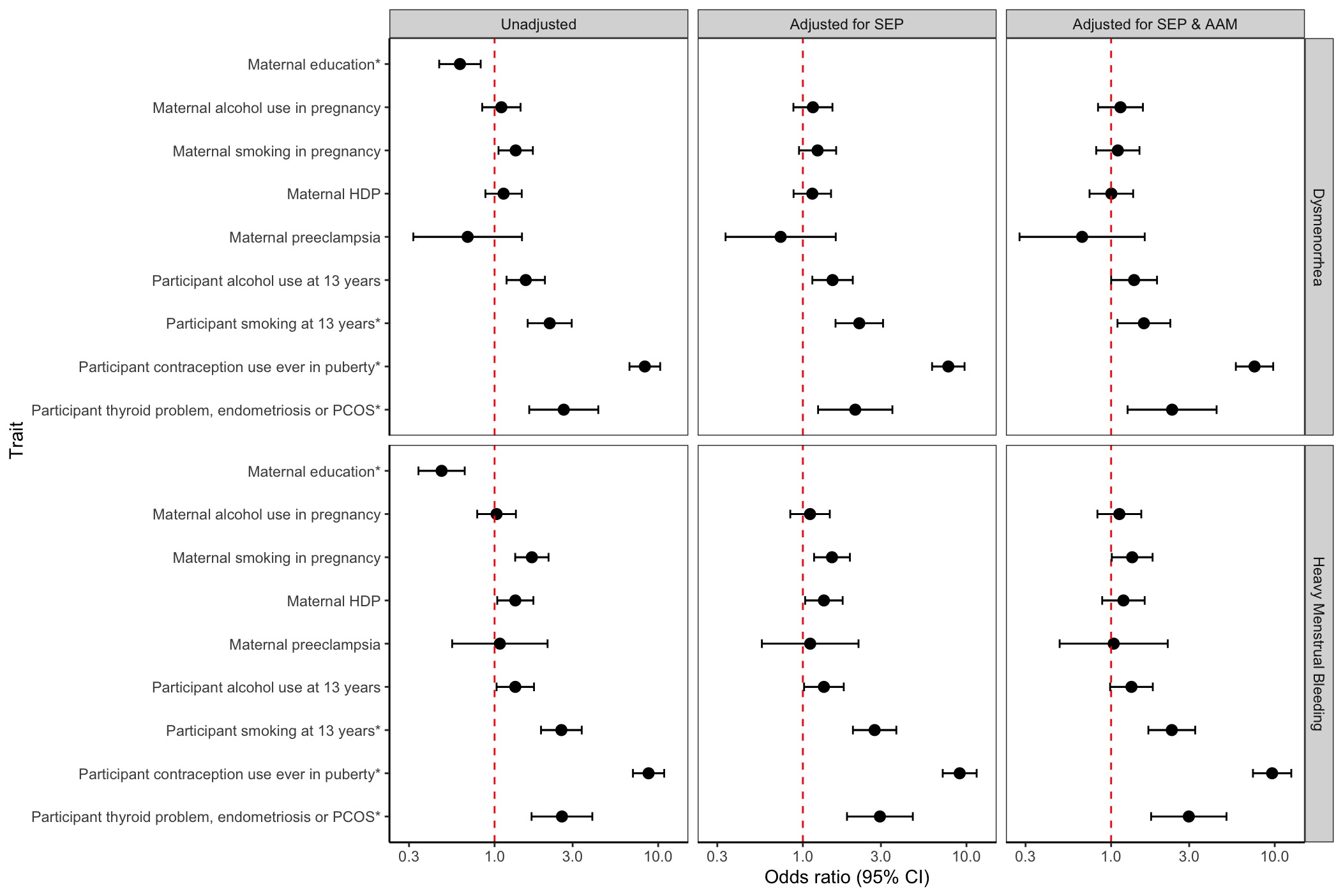

Supplement: Supplementary Figures S1-S8 and Tables S1-S3 [file IEPI_A_2366157_SM0001.zip › Figure S7.jpeg]

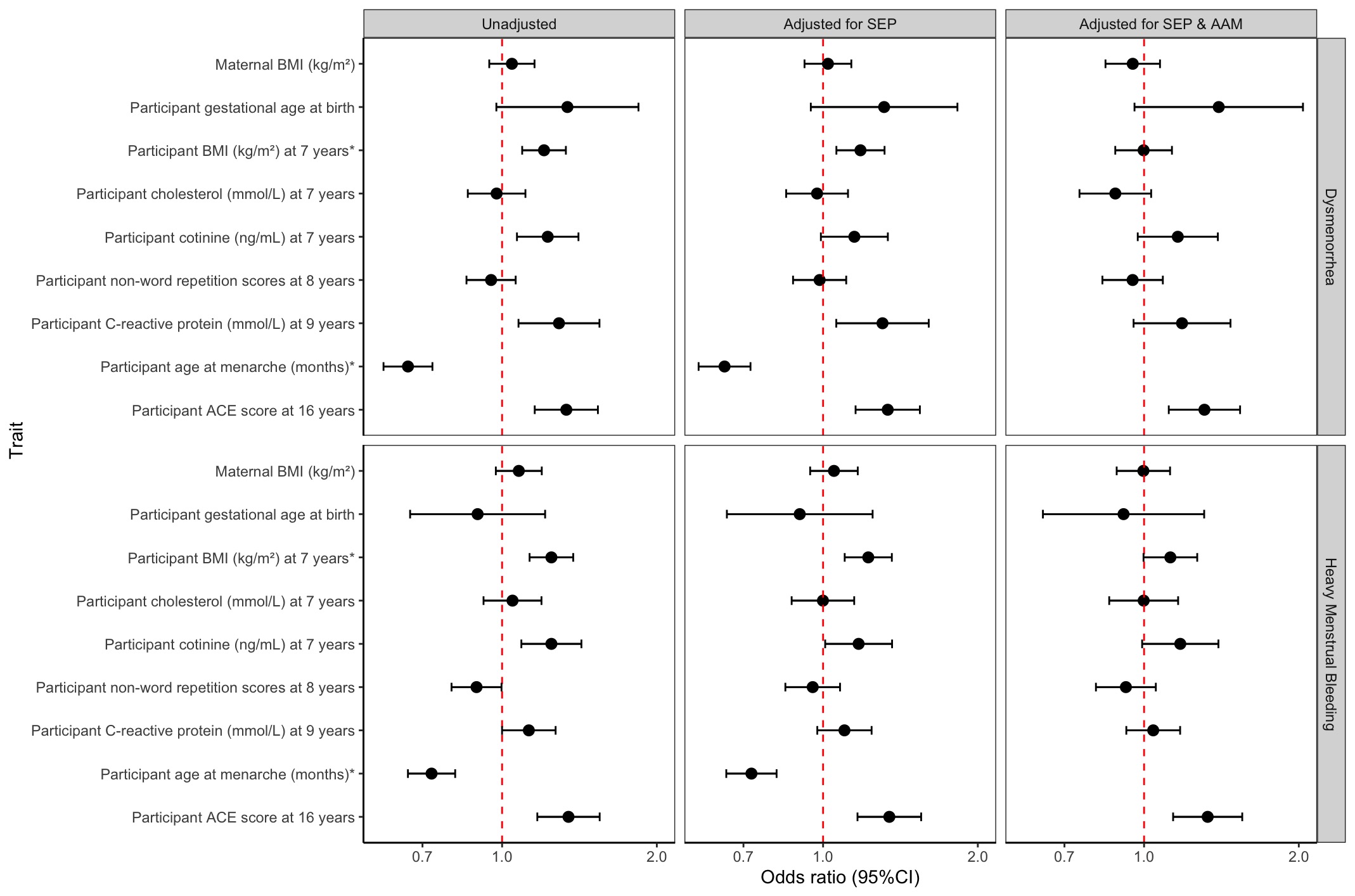

Supplement: Supplementary Figures S1-S8 and Tables S1-S3 [file IEPI_A_2366157_SM0001.zip › Figure S8.jpeg]
